# The Genome of *Nitrospina gracilis* Illuminates the Metabolism and Evolution of the Major Marine Nitrite Oxidizer

**DOI:** 10.3389/fmicb.2013.00027

**Published:** 2013-02-21

**Authors:** Sebastian Lücker, Boris Nowka, Thomas Rattei, Eva Spieck, Holger Daims

**Affiliations:** ^1^Department of Microbial Ecology, Ecology Centre, University of ViennaVienna, Austria; ^2^Department of Microbiology and Biotechnology, Biocenter Klein Flottbek, University of HamburgHamburg, Germany; ^3^Department of Computational Systems Biology, Ecology Centre, University of ViennaVienna, Austria

**Keywords:** *Nitrospina*, nitrite oxidation, nitrification, nitrite-oxidizing bacteria, marine nitrogen cycle, nitrite oxidoreductase

## Abstract

In marine systems, nitrate is the major reservoir of inorganic fixed nitrogen. The only known biological nitrate-forming reaction is nitrite oxidation, but despite its importance, our knowledge of the organisms catalyzing this key process in the marine N-cycle is very limited. The most frequently encountered marine NOB are related to *Nitrospina gracilis*, an aerobic chemolithoautotrophic bacterium isolated from ocean surface waters. To date, limited physiological and genomic data for this organism were available and its phylogenetic affiliation was uncertain. In this study, the draft genome sequence of *N. gracilis* strain 3/211 was obtained. Unexpectedly for an aerobic organism, *N. gracilis* lacks classical reactive oxygen defense mechanisms and uses the reductive tricarboxylic acid cycle for carbon fixation. These features indicate microaerophilic ancestry and are consistent with the presence of *Nitrospina* in marine oxygen minimum zones. Fixed carbon is stored intracellularly as glycogen, but genes for utilizing external organic carbon sources were not identified. *N. gracilis* also contains a full gene set for oxidative phosphorylation with oxygen as terminal electron acceptor and for reverse electron transport from nitrite to NADH. A novel variation of complex I may catalyze the required reverse electron flow to low-potential ferredoxin. Interestingly, comparative genomics indicated a strong evolutionary link between *Nitrospina*, the nitrite-oxidizing genus *Nitrospira*, and anaerobic ammonium oxidizers, apparently including the horizontal transfer of a periplasmically oriented nitrite oxidoreductase and other key genes for nitrite oxidation at an early evolutionary stage. Further, detailed phylogenetic analyses using concatenated marker genes provided evidence that *Nitrospina* forms a novel bacterial phylum, for which we propose the name *Nitrospinae*.

## Introduction

Nitrite-oxidizing bacteria (NOB) are chemolithoautotrophic organisms catalyzing the oxidation of nitrite to nitrate, which is the second step of nitrification and a major biogeochemical process. Nitrite is substrate not only for nitrite oxidation but also for denitrification and anaerobic ammonium oxidation (anammox), both leading to nitrogen losses from ecosystems to the atmosphere. Therefore, the activity of NOB is a key factor controlling the retention of fixed nitrogen in the form of nitrate in the biosphere.

NOB are a phylogenetically diverse functional group. Known representatives belong to the genera *Nitrobacter*, *Nitrotoga*, and *Nitrococcus* within the *Alpha*-, *Beta*-, and *Gammaproteobacteria*, respectively (Winogradsky, 1890; Watson and Waterbury, 1971; Alawi et al., 2007), to the genus *Nitrospira* within the *Nitrospirae* (Daims et al., 2001), and to the genus *Nitrolancetus* within the *Chloroflexi* (Sorokin et al., 2012). The genus *Nitrospina* was provisionally assigned to the *Deltaproteobacteria* (Teske et al., 1994), but this affiliation has been questioned due to difficulties to classify this genus solely by 16S rRNA-based phylogenetic inference (Schloss and Handelsman, 2004). Most NOB lineages are highly versatile and can be found in terrestrial, limnic, and marine ecosystems as well as in man-made habitats such as wastewater treatment facilities. However, the dominant nitrite oxidizers in the oceans appear to be *Nitrospina*. The type species of this genus, *Nitrospina gracilis*, was isolated from ocean surface waters (Watson and Waterbury, 1971). It forms long, slender, Gram-negative rods, grows purely chemoautotrophically with nitrite and CO_2_ as the sole source of energy and carbon, respectively, and was described to be strictly aerobic using oxygen as terminal electron acceptor. No heterotrophic potential was observed and many organic substrates even inhibited the growth of this organism (Watson and Waterbury, 1971). *Nitrospina*-like bacteria were frequently detected by molecular methods in open ocean water (Fuchs et al., 2005; DeLong et al., 2006) as well as in marine sediments (Hunter et al., 2006; Jorgensen et al., 2012), but surprisingly also in marine oxygen minimum zones (OMZs; Labrenz et al., 2007; Fuchsman et al., 2011) and anoxic marine sediments (Davis et al., 2009; Jorgensen et al., 2012). Moreover, nitrite oxidation has been measured in OMZs (Füssel et al., 2012). Interestingly, the distribution profiles of *Nitrospina* and ammonia-oxidizing archaea (AOA) were found to correlate in some coastal and open ocean habitats (Mincer et al., 2007; Santoro et al., 2010). Prolonged coexistence of *Nitrospina* and AOA closely related to “*Candidatus* Nitrosopumilus maritimus” was even observed in an enrichment culture (Park et al., 2010), further strengthening the functional link between these nitrifiers. The only non-marine habitats *Nitrospina*-like 16S rRNA sequences were derived from are uranium mill tailings (Radeva and Selenska-Pobell, 2005) and an alpine subsurface radioactive thermal spring (Weidler et al., 2007). Thus, *Nitrospina* apparently are confined mainly to marine environments.

In marine ecosystems, nitrate accounts for as much as 88% of the fixed nitrogen, corresponding to 5.8 × 10^5^ Tg (Gruber, 2008). While rapidly assimilated by phytoplankton in ocean surface waters, nitrate accumulates in the deep sea where it constitutes the largest pool of fixed inorganic nitrogen in the biosphere. The only biochemical reaction known to form nitrate is bacterial nitrite oxidation, which takes place during nitrification and, to a lesser extent, during anammox (Strous et al., 2006). Contrasting the obviously immense importance of *Nitrospina* as a major source of nitrate in marine environments, very little biochemical and genomic data is available for these enigmatic organisms, and even the phylogenetic affiliation of the genus is still unclear. This embarrassing lack of knowledge is mainly caused by the recalcitrance of *Nitrospina* to cultivation in the laboratory. Their slow growth rates with a minimum generation time of 24 h (Watson and Waterbury, 1971) further impede biochemical characterization. In this study, we employed a genomic approach to gain deeper insights into the metabolic potential of *Nitrospina gracilis* strain 3/211. The sequenced genome of this organism shed light on the core metabolism enabling *Nitrospina* of chemolithoautotrophic growth with nitrite. Comparative genomic analyses illuminated the evolutionary descent of *Nitrospina* and its nitrite-oxidizing capacity. By utilizing concatenated datasets of established marker proteins, the phylogenetic affiliation of *Nitrospina* could finally be assessed.

## Materials and Methods

### Cultivation

Cells of *N*. *gracilis* were grown in 5 l batch cultures in mineral salts medium prepared with 70% seawater containing 0.3 mM nitrite as the sole energy source. The marine medium (Watson and Waterbury, 1971) was composed of 700 ml filtered seawater and 300 ml distilled water, supplemented with 0.005 g l^−1^ CaCl_2_ × 2 H_2_O, 0.1 g l^−1^ MgSO_4_ × 7 H_2_O, 0.001 g l^−1^ FeSO_4_ × 7 H_2_O, 0.0017 g l^−1^ KH_2_PO_4_, 6 μg l^−1^ CuSO_4_ × 5 H_2_O, 25 μg l^−1^ Na_2_MoO_4_ × 2 H_2_O, 50 μg l^−1^ MnCl_2_ × 4 H_2_O, 0.5 μg l^−1^ CoCl_2_ × 6 H_2_O, and 25 μg l^−1^ ZnSO_4_ × 7 H_2_O. The pH was adjusted to 6.5–7.0 and changed to 7.4–7.6 within 2 days after autoclaving. Cultures were started with 1% inoculum and incubated at 28°C in the dark. When nitrite was consumed for the first time, the culture bottles were started to be stirred moderately. Nitrite consumption was measured and substrate was replenished regularly. To check for purity of the culture, aliquots were incubated in heterotrophic marine medium (0.15 g l^−1^ bactopeptone, 0.15 g l^−1^ yeast extract, 0.055 g l^−1^ sodium pyruvate in marine mineral salts medium) without nitrite.

### DNA extraction, genome sequencing, and annotation

High-molecular weight genomic DNA was isolated following the hexadecyltrimethylammonium bromide (CTAB) protocol as provided by the DOE Joint Genome Institute (JGI, http://my.jgi.doe.gov/general/protocols.html) with minor modifications. Shortly, cells were resuspended in TE buffer and digested by the sequential addition of lysozyme and 10% (w/v) sodium dodecylsulfate (SDS) and Proteinase K. Following the subsequent incubation in CTAB/NaCl solution, the DNA-containing aqueous phase was extracted in Roti-Phenol/Chloroform/Isoamylalcohol (Carl Roth, Karlsruhe, Germany) and washed in Roti-C/I once to remove residual phenol. After DNA precipitation with isopropanol and washing with 70% ethanol, the DNA was suspended in TE buffer and stored at −20°C. Genome sequencing was performed at LGC Genomics (Berlin, Germany) using GS FLX Titanium sequencing technology. One 1/4 picotiter plate was run with an 8 kb paired-end large-insert library. Sequence read filtering and correction, contig assembly, and scaffold formation were done by LGC Genomics.

The draft genome of *N. gracilis* was integrated into the MicroScope annotation platform (Vallenet et al., 2009). After automated prediction and annotation of coding sequences (CDS), the annotation of all CDS in key pathways, including those for nitrite oxidation, respiration, and carbon fixation, was manually refined by using the respective tools of MaGe (Vallenet et al., 2006) as described in detail elsewhere (Lücker et al., 2010).

The draft genome sequence of *Nitrospina gracilis* strain 3/211 has been deposited at EMBL-EBI under project number PRJEB1269.

### Amplification, cloning, and sequencing of *nxrB* genes

As both copies of the operon containing the genes of nitrite oxidoreductase (NXR) were incomplete and lacked the *nxrB* gene coding for the beta subunit of NXR, these regions were specifically PCR-amplified from genomic DNA, cloned in *E. coli* by TOPO-TA Cloning (Life Technologies, Paisley, UK), and Sanger-sequenced (3130xl Genetic Analyzer, Applied Biosystems, Vienna, Austria). Operon-specific PCR was carried out using the newly designed primers Nspn_nxrA1_3064f (CACTCTTGCTGGACGTCA; forward) in combination with Nspn_nxrC1_111r (CATATCCACAACCACGTG; reverse) and Nspn_nxrA2_3064f (CATTCAGCATGGCAGAGC; forward) with Nspn_nxrC2_111r (CAAATCGATCACCACTCC; reverse). These primer pairs target the C-terminal ends of the alpha subunit genes *nxrA* and the N-terminal parts of the gamma subunits *nxrC*. Cycling conditions were as follows: an initial denaturation step (94°C, 5 min) was followed by 35 cycles of template denaturation (94°C, 30 s), primer annealing (52°C, 30 s), and product elongation (72°C, 1.5 min). Cycling was completed by a final elongation step (72°C, 20 min). The cloned amplicons were sequenced using M13 primers.

*nxrB* sequences obtained in this study have been deposited at GenBank under accession numbers KC262217 and KC262218.

### Phylogenetic analyses

An amino acid sequence database of heme-copper oxidase-family enzymes was established using the software ARB (Ludwig, 2004). Multiple sequence alignments were generated automatically using ClustalW2 (Larkin et al., 2007) and manually refined using the sequence editor included in ARB. Protein sequences of the NxrA and NxrB subunits of NXR were imported into existing datasets of type II DMSO reductase-family molybdopterin cofactor-binding proteins (Lücker et al., 2010). The sequences were aligned using the automatic aligner integrated in ARB, followed by manual refinement.

Amino acid sequences of 49 established phylogenetic marker proteins (Table A1 in Appendix) were downloaded from the Microbial Genome Database for Comparative Analysis (Uchiyama et al., 2010). Ortholog clusters were manually filtered to include bacterial sequences only. To avoid erroneous phylogenetic signals caused by horizontal gene transfer (HGT) or incorrect annotations, organisms with more than one entry for a query protein were excluded. This resulted in datasets with 443–502 entries for each protein. The single-protein datasets were automatically aligned with ClustalO (Sievers et al., 2011), running two iterations, followed by filtering for badly aligned regions or sites of rare insertions by Gblocks (Talavera and Castresana, 2007) with settings “Minimum Length of an Initial Block (−b0) = 2,” “Maximum Number of Contiguous Non-conserved Positions (−b3) = 50,” “Minimum Length of A Block (−b4) = 3,” and “Allowed Gap Positions (−b5) = all” to obtain less stringent filtering. The filtered alignment files were subsequently concatenated using an in-house generated C++ program and imported into ARB.

Phylogenetic analyses were performed by applying maximum-likelihood, maximum-parsimony, and Bayesian interference methods: RAxML [version 7.0.3 (Stamatakis et al., 2005); 100 rapid or 100 advanced bootstrap iterations], PhyML (Felsenstein, 2005), ProML and ProtPars [PHYLIP package, version 3.6 (Guindon and Gascuel, 2003); 100 bootstrap iterations], TreePuzzle [version 5.0 (Schmidt et al., 2002)], and MrBayes [version 3.1 (Ronquist and Huelsenbeck, 2003); 2–5 M generations] using the JTT, WAG, or Blosum62 substitution models. Where applicable, N-terminal signal peptide sequences were excluded from the analyses and manually created indel filters were used.

### Phylome construction

For the calculation of phylogenetic trees for each protein of the *in silico* deduced proteome of *N. gracilis*, the software PhyloGenie (Frickey and Lupas, 2004) was used as previously described (Lücker et al., 2010). The reference database for PhyloGenie was generated from the National Center for Biotechnology Information non-redundant protein database NCBI nr (Sayers et al., 2011), in which taxon names were edited to remove characters that would interfere with the export of phylogenetic trees in the Newick file format. The NCBI taxonomy database name file was adapted in a similar manner. PhyloGenie analysis was performed for each query protein using the default parameters with the following modification: −blammerparams = −taxid f. For the BLAST (Altschul et al., 1990) searches in PhyloGenie, NCBI BLAST (version 2.2.19) was used. Protein phylogenies were calculated on the basis of full or partial automatic alignments produced by the BLAMMER program included in PhyloGenie. Trees were calculated using RAxML (Stamatakis et al., 2005) with the JTT substitution model and 100 bootstrap iterations. All trees were subject of specific taxonomic queries using Phat (Frickey and Lupas, 2004). Furthermore the taxonomy of closest homologs was determined from these trees since this strategy is less prone to alignment artifacts than best hits from automatic multiple sequence alignments. An in-house Perl script determined the tree node closest to the respective query proteins from *N. gracilis* and consecutively sorted all operational taxonomic units behind this node by their distances to the query. The organism with the smallest distance was considered to contain the closest homologous protein.

## Results

### Genome reconstruction

The obtained sequence reads were assembled into 109 contigs, which were arranged in four scaffolds. The largest scaffold encompassed 106 contigs, whereas the remaining three scaffolds consisted of single contigs whose location relative to the other contigs could not be determined unambiguously. The presence of frameshift mutations in some essential and highly conserved genes indicates that some pyrosequencing-associated errors could not be detected and corrected during read filtering and assembly. The obtained genome sequence of *N. gracilis* has a size of 3,067,213 bp and an average G + C content of 56.2%. It contains 3,147 predicted coding sequences (CDS; Table 1), but the large number of gene fragments (due to sequence gaps within genes and frameshifts) likely causes an overestimation of the CDS count. The genome sequence contains 1 complete *rrn* operon and 45 tRNA genes (1–5 for each of the 20 amino acids). The sequenced genome is near-complete based on the full set of tRNAs, the presence of all 127 clusters of orthologous groups (COGs) of proteins conserved in all 50 bacterial genomes currently in the COG database (Tatusov et al., 2003), and the estimated small gap sizes within the large scaffold.

**Table 1:** **Overview of key features of the *Nitrospina gracilis* strain 3/211 genome**.

Draft genome size		3,067,213 bp	
Estimated genome size		3,101,305 bp	
Estimated genome completeness		98.90%	
Number of scaffolds		4	
Number of contigs		109	
Average G + C content		56.21%	
Number of genomic objects [CDS, fragment CDS (r,t)RNA]		3202	
Number of coding sequences (CDS)		3147	
rRNA genes		3	
tRNA genes		45	

**Functional category**		**CDS**	**CDS (%)**

**CLUSTERS OF ORTHOLOGOUS GROUPS (COG) AUTOMATED CLASSIFICATION**			
D	Cell cycle control, cell division, chromosome partitioning	43	1.37
M	Cell wall/membrane/envelope biogenesis	222	7.05
N	Cell motility	97	3.08
O	Posttranslational modification, protein turnover, chaperones	176	5.59
T	Signal transduction mechanisms	166	5.27
U	Intracellular trafficking, secretion, and vesicular transport	91	2.89
V	Defense mechanisms	59	1.87
A	RNA processing and modification	1	0.03
B	Chromatin structure and dynamics	1	0.03
J	Translation, ribosomal structure, and biogenesis	181	5.75
K	Transcription	114	3.62
L	Replication, recombination and repair	140	4.45
C	Energy production and conversion	219	6.96
E	Amino acid transport and metabolism	295	9.37
F	Nucleotide transport and metabolism	79	2.51
G	Carbohydrate transport and metabolism	138	4.39
H	Coenzyme transport and metabolism	114	3.62
I	Lipid transport and metabolism	78	2.48
P	Inorganic ion transport and metabolism	196	6.23
Q	Secondary metabolites biosynthesis, transport, and catabolism	94	2.99
R	General function prediction only	456	14.49
S	Function unknown	238	7.56

Notably, the smallest of the four scaffolds contained only an *nxrB* gene coding for the beta subunit of nitrite oxidoreductase (NXR). The NXR operon, which contains the genes of the alpha (*nxrA*), beta (*nxrB*), and gamma (*nxrC*) subunits, is duplicated in the *N. gracilis* genome. Since the sequences of the two *nxrB* genes are highly similar, the complete NXR operons could not be unambiguously assembled from the sequence reads. Therefore, operon-specific primers where designed and used to amplify the genomic regions between *nxrA1* and *nxrC1*, and *nxrA2* and *nxrC2*, respectively, in order to obtain by cloning and Sanger-sequencing the complete operon sequences including *nxrB*. When translated into their protein sequence, the two NxrB copies had a length of 425 amino acids and an identity of 100% (with five synonymous single-base substitutions on the nucleotide level).

### Phylogeny

**Figure 1:**
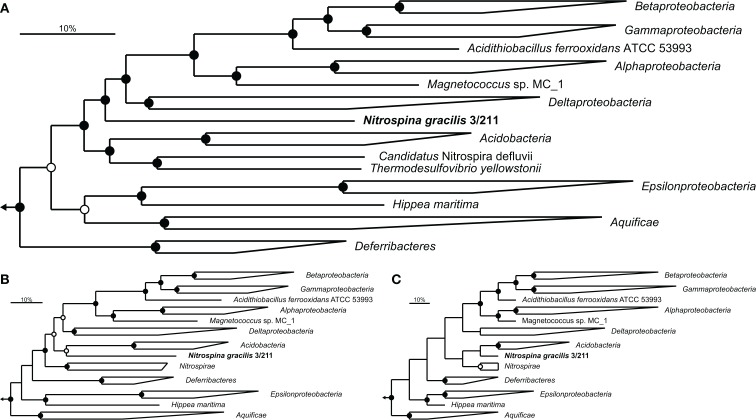
**Phylogenetic affiliation of *N*. *gracilis* 3/211 (boldface)**. Trees were calculated using a concatenated dataset of 49 proteins (Table A1 in Appendix). Members of the phylum *Planctomycetes* were used as outgroup. Filled and open circles represent statistical support ≥90 and >75%, respectively. The scale bar represents 10% estimated sequence divergence. **(A)** Bayesian inference tree (SD = 0.009985) run for 8 M generations. In total, 6962 alignment positions were considered. **(B,C)** Maximum-likelihood and maximum-parsimony analysis, respectively, with 100 bootstrap iterations, using 7629 alignment positions.

A set of 49 established phylogenetic marker proteins (Table A1 in Appendix) was extracted from the genome of *N. gracilis* and a range of other complete bacterial genomes covering all bacterial phyla for which genome sequences are available. The concatenated dataset of these proteins enabled a detailed phylogenetic analysis of the affiliation of *Nitrospina* within the *Bacteria*. Preliminary trees containing sequence information from 502 bacteria indicated deep phylogenetic branching of *N. gracilis* between the *Proteobacteria* and a group consisting of the *Acidobacteria* and *Nitrospirae* phyla (data not shown). Consecutively, a reduced dataset containing representatives of these and few other closely affiliated phyla was generated and used for more detailed analyses employing different tree calculation methods. These analyses clearly indicated a position of *N. gracilis* close to, but outside the *Proteobacteria* (Figure 1) and not within any other bacterial phylum either. Notably, the *Epsilonproteobacteria* did not cluster with the other *Proteobacteria*, a branching pattern that has been observed previously (Ludwig, 2010), and formed a separate branch together with the *Aquificae*. This grouping of *Aquificae* and *Epsilonproteobacteria* is consistent with other marker protein-based analyses that also found the *Aquificae* not to group as one of the most basal bacterial phyla (Korbel et al., 2002; Cavalier-Smith, 2006). The *Acidobacteria* and *Nitrospirae* formed a superphylum in the Bayesian interference analysis that grouped as the next most closely related phyla to *Nitrospina* (Figure 1A), followed by the aforementioned *Aquificae*/*Epsilonproteobacteria* group and the *Deferribacteres*. Maximum-likelihood analysis grouped *Nitrospina* as sister phylum of the *Acidobacteria*, with the *Nitrospirae* forming a separate branch (Figure 1B), and the maximum-parsimony approach indicated a superphylum of all three groups (Figure 1C). The separation of *Nitrospina* from the *Proteobacteria* thus is supported by all analyses, and the grouping with the *Acidobacteria* and *Nitrospirae* in some analyses strongly contradicts the possibility that *Nitrospina* forms a basal new class within the *Proteobacteria*.

To further assess the separate phylogenetic position of *N. gracilis* as member of a novel phylum, two additional methods were employed: comparison of shared conserved insertion and deletion signature sequences (indel analysis; Gupta and Griffiths, 2002) and gene transposition analysis (Kunisawa, 2006). Both analyses were consistent with the calculated phylogenetic trees by suggesting that *N. gracilis* belongs to a phylum related to, but separate from the *Proteobacteria*. *N. gracilis* carries the characteristic insertions in the transcription termination factor Rho and the alanine-tRNA ligase AlaS, which are common to all *Proteobacteria* and a range of phyla including the *Chlamydiae* and *Aquificae* (Gupta, 2000; Gupta and Griffiths, 2002), but lacks signature sequences in the inorganic phosphatase Ppi, molecular chaperone DnaK, and CTP synthetase PyrG that distinguish the *Proteobacteria* from the other phyla (Gupta, 2000, 2001). Analyses of gene transposition events (Kunisawa, 2010, 2011) also indicated a close relationship to the *Proteobacteria*, *Nitrospirae*, and to a lesser extent the *Deferribacteres* and *Aquificae*, but clearly distinguished *N. gracilis* from these established phyla based on unique differences in bordering genes.

**Figure 2:**
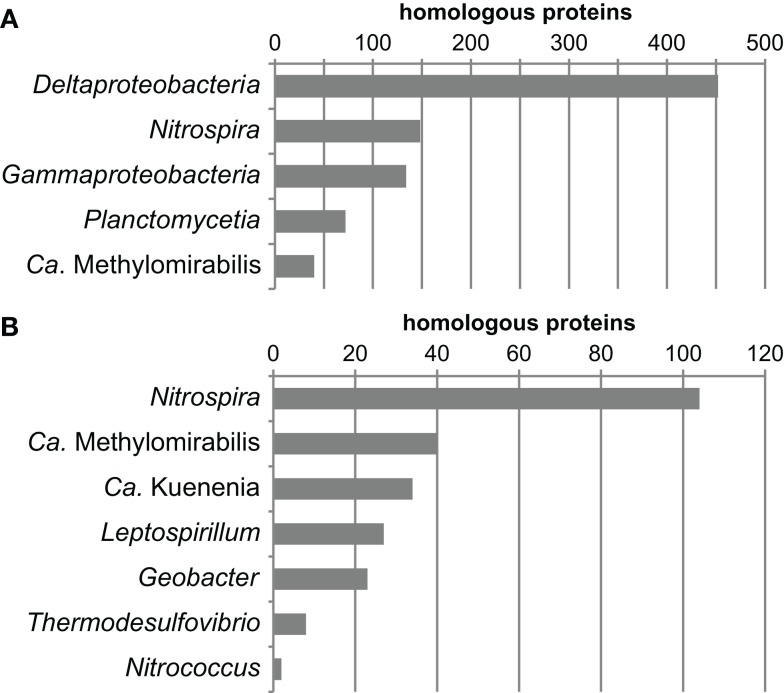
**Summarized results of a phylogenetic analysis of the phylome of *N. gracilis* 3/211**. The nearest phylogenetic neighbor (closest homolog) in other sequenced genomes was determined for each protein of *N. gracilis*. The resulting trees were queried for the closest homolog using Phat (Frickey and Lupas, 2004). The graphs depict the phylogenetic groups displaying the largest numbers of most closely related homologs on the **(A)** class and **(B)** genus level. In total, Phat could unambiguously assign closest homologs for 2,246 proteins.

Another way to look at ancestry and identify HGT events at the same time is by phylome reconstruction, which is the determination of the relationship of every protein encoded in a genome by phylogenetic inference (Frickey and Lupas, 2004). If the exact phylogenetic position of an organism is known this is an elegant method to determine the fraction of horizontally transferred genes and to identify potential donor organisms. For organisms of uncertain affiliation, the phylomic information may reveal ancestral relationships since most genes are inherited vertically. Therefore, the phylome of *N. gracilis* was reconstructed and the closest relative (based on the distance to the respective homolog in *N. gracilis*) was extracted from each single-protein tree. Of the 2,354 proteins of *N. gracilis* that have homologs in public databases, a large part clustered on the class level with *Deltaproteobacteria* (*n* = 514), followed by *Gammaproteobacteria* (*n* = 225) and *Nitrospira* (*n* = 211). On the genus level *Nitrospira* had most hits (*n* = 161), followed by the deltaproteobacterial *Geobacter* (*n* = 81), the recently described “*Candidatus* Methylomirabilis” (Ettwig et al., 2010; *n* = 68), and “*Candidatus* Kuenenia” (Strous et al., 2006; *n* = 51). The numerous closest homologs in *Proteobacteria* and *Nitrospira* are consistent with the phylogenetic trees as well as the indel and gene transposition analyses. The large number of proteins clustering with *Nitrospira* may also reflect a high degree of functional similarity in *Nitrospina* and *Nitrospira*. In contrast, the large number of proteins affiliated to homologs in *Methylomirabilis* and *Kuenenia* was unexpected and might indicate a large extent of HGT between these organisms and *Nitrospina*. One possible caveat of the applied tree query approach is that the next neighbors in a tree may not always have the shortest distance to each other. However, manual reassessment by using Phat within the PhyloGenie program (Frickey and Lupas, 2004) revealed the same trends for the relationship of *Nitrospina* proteins to the aforementioned groups (Figure 2). These results strongly corroborate the phylogenetic placement of *Nitrospina* in a distinct phylum with the *Proteobacteria* and *Nitrospirae* as most closely related known phyla.

### Nitrite oxidation and nitrogen metabolism

*N. gracilis* is a chemolithoautotrophic nitrite-oxidizing bacterium. It gains all energy required for growth from the oxidation of nitrite and fixes CO_2_ as sole carbon source, using electrons derived from nitrite oxidation. The key enzyme for nitrite oxidation is nitrite oxidoreductase, which shuttles two electrons per oxidized ${\text{NO}}_{2}^{-}$ into the electron transport chain. The NXR belongs to the type II group of molybdopterin-binding enzymes within the DMSO reductase enzyme family (Jormakka et al., 2004). These enzymes are membrane-associated complexes of three subunits. The catalytic alpha subunit contains the molybdenum *bis* molybdopterin guanine dinucleotide (Mo-*bis*-MGD) cofactor and one [4Fe–4S] iron-sulfur (Fe-S) cluster. The beta subunit contains one [3Fe-4S] and three [4Fe-4S] cluster and transfers the electrons from the alpha subunit to the membrane-integral gamma subunit (Martinez-Espinosa et al., 2007). This membrane subunit functions as membrane anchor of the holoenzyme and channels the electrons to and from the electron transport chain *via* one or two hemes (Rothery et al., 2008).

*N. gracilis* contains two operons encoding putative NXR, which consist of the genes coding for all three subunits in an *nxrABC* order (Table S1 in Supplementary Material). The two operon copies are highly similar, with an overall identity of 94.6% on the nucleotide level. When translated into their protein sequences, the α (NxrA), β (NxrB), and γ (NxrC) subunits have identities of 94.9, 100, and 87.5%, respectively. NxrA1 and NxrA2 contain an N-terminal twin-arginine motif for export into the periplasmic space *via* the twin-arginine protein translocation (Tat) pathway. Both NxrB copies lack a signal peptide but may be co-translocated into the periplasm with the large subunit by a “hitchhiker” mechanism by the Tat pathway as proposed for “*Candidatus* Nitrospira defluvii” (Lücker et al., 2010) and observed for other members of the DMSO reductase-family of molybdopterin-binding enzymes (Martinez-Espinosa et al., 2007). The two γ subunits encoded in the NXR operons are predicted by Pfam (Finn et al., 2010) to contain a DMSO reductase-like heme *b*-binding domain and have a predicted N-terminal signal peptide that overlaps with a predicted single transmembrane helix. The Phobius (Käll et al., 2007) and SignalP 4.0 (Petersen et al., 2011) prediction methods favor secretion of NxrC into the periplasm *via* the Sec protein tanslocation pathway. This is puzzling, since NxrC of *Ca*. N. defluvii and the *Nar*I homologs in NarG-like molybdopterin-binding enzymes are membrane-integral (Rothery et al., 2008; Lücker et al., 2010). It implies that NxrC of *N*. *gracilis* is a soluble heme *b* subunit as found in selenate (Ser) and chlorate (Clr) reductases (Bäcklund et al., 2009; Lowe et al., 2010) and ethylbenzene (Ebd) and dimethylsulfide (Ddh) dehydrogenases (Kniemeyer and Heider, 2001; McDevitt et al., 2002). These enzymes form soluble periplasmic protein complexes and interact with soluble cytochrome *c* proteins, which shuttle electrons to and from the membrane-integral respiratory chain (Lowe et al., 2010). In *Nitrospina*, however, the NXR complex is membrane-associated (Bartosch et al., 1999; Spieck and Bock, 2005b). Thus, an additional membrane anchor likely is required for the formation of active NXR in this organism. In addition to the two linked copies of NxrC, *N. gracilis* has genes of two other putative γ-subunits (Table S1 in Supplementary Material). NxrC3 is located in the direct vicinity of a TorD-like chaperone that most likely functions in Mo cofactor insertion into NxrA prior to Tat export, as has been proposed for other Mo-*bis*-MGD-containing enzymes such as selenate reductase (Guymer et al., 2009). This NxrC has one predicted membrane helix as well as the DMSO reductase-like heme *b*-binding domain. NxrC4 is encoded in a genetic context unrelated to nitrite oxidation and shows only low similarities to the other NxrC copies, but also contains one predicted transmembrane helix and the heme *b*-binding domain. Aside from these candidate NXR γ subunits, the genome of *N. gracilis* codes for at least one putative alternative NxrC (alt_NxrC1, Table S1 in Supplementary Material). This protein is remarkable since it contains not only two transmembrane helices, but also two cytochrome *c*-type heme *c* binding sites. The heme *c* groups might have an important function in nitrite oxidation, since cytochrome *c* is predicted to be the primary acceptor of the high-potential electrons derived from nitrite in NOB ($E^{0^{\prime }}=+430\text{mV}$; Sundermeyer-Klinger et al., 1984). Thus, electrons might be channeled through NxrB and possibly NxrC to the alt_NxrC subunit, where the *c*-type hemes could shuttle the electrons to acceptors downstream in the respiratory chain. The genome contains three additional copies of this putative alternative γ subunit (Table S1 in Supplementary Material), which all contain 1–3 cytochrome *c* binding sites and an additional DMSO reductase-like heme *b*-binding domain. However, alt_NxrC2 and 4 are predicted to be periplasmic rather than membrane-integral and might function as soluble electron transfer proteins as described for cytochrome *c*_4_ and selenate reductase (Lowe et al., 2010). Future studies may resolve the true subunit composition of the NXR complex in *Nitrospina*.

**Figure 3:**
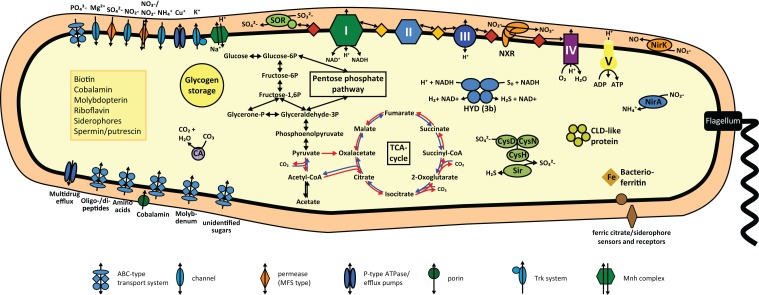
**Cell metabolic cartoon based on the annotation of the *N. gracilis* 3/211 genome**. CLD, chloride dismutase; CA, carbonic anhydrase; CysDNC, sulfate adenylyltransferase/adenylylsulfate kinase; HYD, hydrogenase; NirA, ferredoxin-nitrite reductase; NirK, Copper-containing nitrite reductase; NXR, nitrite oxidoreductase; Sir, ferredoxin-sulfite reductase; SOR, Sulfite:cytochrome c oxidoreductase. Enzyme complexes of the electron transport chain are labeled by Roman numerals. Red and orange diamonds represent cytochrome *c* proteins and quinones, red and blue arrows the oxidative and reductive TCA cycle, respectively.

The *N*. *gracilis* genome encodes two copies of copper-containing nitrite reductase NirK (Figure 3; Table S1 in Supplementary Material). NirK1 contains all conserved amino acid residues required for binding of the two Cu(II) sites and of nitrite. In the second copy NirK2 the weak ligand of the type 1 copper center methionine is mutated to lysine. NirK1 thus is expected to be a functional nitrite reductase, while NirK2 might have reduced or altered activity (Wherland et al., 2005). Interestingly, all analyzed genomes of NOB except *Nitrolancetus hollandicus* (Sorokin et al., 2012) contain *nirK* genes, but the function of nitrite reduction in NOB has not clearly been determined yet. No known nitric oxide (NO) reductase is encoded in the *N*. *gracilis* genome and a role of NirK in denitrification therefore seems unlikely. NO stimulated NADH formation in *Nitrobacter* and was speculated to be an intermediate of nitrite oxidation in this organism (Freitag and Bock, 1990; Poughon et al., 2001). A later study proposed an alternative role of NO in the regulation of the cellular redox state in *Nitrobacter* (Starkenburg et al., 2008). In this model the terminal cytochrome *c* oxidase is reversibly inhibited by NO under low-oxygen conditions, leading to an increased flow of electrons through the reverse electron transport chain. This causes higher CO_2_ fixation rates and may increase the biosynthesis of the storage compound poly-β-hydroxybutyrate (Starkenburg et al., 2008). For NOB other than *Nitrobacter* no function of NirK has been proposed, and denitrification of nitrite to NO under anoxic conditions has not been observed yet.

*N. gracilis* grows on nitrite as sole source of both energy and nitrogen. Consistently, the genome contains the *nirA* gene for assimilatory ferredoxin-nitrite reductase, which is located directly next to *nirC* encoding a nitrite transporter of the formate/nitrite transporter family (Figure 3; Table S1 in Supplementary Material). A second nitrite transporter of the NarK nitrate/nitrite antiporter type, as well as two Amt-type ammonium transporters, likely is also involved in nitrogen uptake for assimilation (Table S1 in Supplementary Material).

### Energy metabolism and reverse electron transport

**Figure 4:**
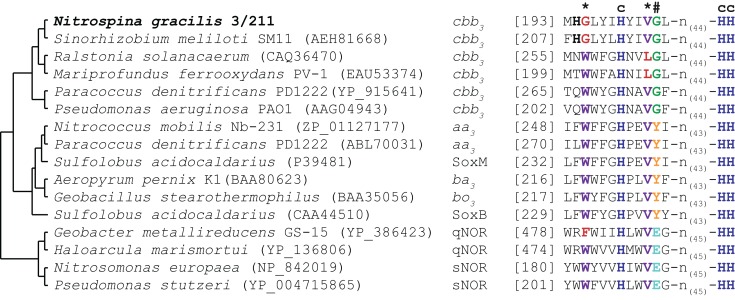
**Partial multiple sequence alignment of selected heme-copper oxidase-family members**. Relationship of the enzymes is indicated by a cladogram. The oxidase type is indicated for each enzyme. Amino acid residues involved in the formation of the copper (Cu_B_)-binding site are highlighted in color and by a symbol indicating function: dark blue, histidine residues involved in Cu_B_-binding (c); purple, residues involved in electron transport from the high-spin heme to Cu_B_(*), red, uncommon amino acids; orange, crosslinking tyrosine residue stabilizing one of the Cu_B_-binding histidines (#), green and turquoise, alternative conserved residues in this position.

*N. gracilis* encodes a terminal oxidase of the cytochrome *cbb*_3_-type (Figure 3; Table S1 in Supplementary Material). As the *cbb*_3_-type terminal oxidases have high affinities for O_2_ (Cosseau and Batut, 2004), its presence will allow *Nitrospina* to sustain respiration in low-oxygen environments. The electrons derived from nitrite oxidation most likely are shuttled *via* cytochrome *c* to this terminal oxidase for reduction of O_2_ as terminal electron acceptor, yielding the energy for the formation of a proton gradient across the cytoplasmic membrane. The proton motive force drives ATP formation by a canonical F_1_F_0_ ATPase (Figure 3; Table S1 in Supplementary Material). Interestingly, the three subunits of the cytochrome *cbb_3_* oxidase of *Nitrospina* are fused into one gene encoding a protein of 954 aa. The N-terminal part of this protein corresponds to subunit I, has 13 predicted transmembrane helices, and contains most of the characteristic binding sites for two heme *b* and the low-potential copper (Cu_B_) center. The C-terminal part corresponds to subunits II and III with one and two heme *c* binding sites, respectively. Interestingly, one amino acid replacement in the Cu_B_ center is unprecedented in heme-copper oxidases (Figure 4). In all members of this family the copper is complexed by three histidine residues, which are numbered His^207^, His^257^, and His^258^ in the cytochrome *cbb_3_* oxidase of *Pseudomonas stutzeri* whose structure has been determined (Buschmann et al., 2010). At position 203 (*P. stutzeri* numbering) most of these enzymes contain a highly conserved tryptophan that is involved in passing electrons from the high-spin heme *b_3_* to the Cu_B_ center where oxygen is reduced (Zaslavsky and Gennis, 2000; Pereira et al., 2001). This tryptophan is replaced by glycine in the *N. gracilis* enzyme, thus clearly impeding any charge transfer. Directly upstream, however, is an additional histidine residue found only in *N. gracilis* (His^202^; *P. stutzeri* numbering) whose positively charged side chain may adopt the role of the hydrophobic Trp^203^ in electron transfer. In addition, two uncommon tyrosine residues flanking the His^207^ at positions 205 and 208 (*P. stutzeri* numbering) might also function as electron carriers. Besides *Nitrospina*, the His^202^, Gly^203^, and Tyr^208^ residues are found only in subunit I of putative heme-copper oxidases from various *Sinorhizobium* genomes (e.g., *Sinorhizobium meliloti* SM11, Figure 4), where they occur in one of several homologous cytochrome *cbb_3_* oxidases. Their presence in other organisms strongly suggests that these unusual amino acid substitutions are not sequencing errors in the *N. gracilis* genome.

*Nitrospina* depends on the electrons derived from nitrite oxidation for the regeneration of reducing equivalents and for autotrophic CO_2_ fixation by the reductive tricarboxylic acid (rTCA) cycle. These electrons enter the respiratory chain at the level of cytochrome *c* due to the high redox potential of the ${\text{NO}}_{2}^{-}$/${\text{NO}}_{3}^{-}$ couple. Therefore, reverse electron transport to NAD^+^ ($E^{0^{\prime }}=-320\text{mV}$) and to ferredoxin ($E^{0^{\prime }}\leq -500\text{mV}$) is required and most likely powered by the proton motive force across the cytoplasmic membrane. Mechanistically, the cytochrome *bc* complex and NADH-quinone oxidoreductase (complexes III and I of the respiratory chain, respectively; Elbehti et al., 2000) are involved in electron transport from cytochrome *c* to NAD^+^, whereas a novel type of complex I may catalyze the reduction of ferredoxin. In addition, electrons can be shuttled from the quinone pool into the rTCA cycle by the succinate dehydrogenase/fumarate reductase (complex II; Lemos et al., 2002). Moreover, the use of organic storage compounds such as glycogen as energy source requires a respiratory chain for the forward transport of electrons. Genome-based hypotheses on forward and reverse electron transport in *N. gracilis* are outlined in the following paragraphs, in the order of electron flow from cytochrome *c* to NAD^+^ and ferredoxin.

The canonical cytochrome *bc* complex in bacteria is composed of three subunits, a Rieske-type [2Fe-2S] subunit, a cytochrome *b* subunit that contains the quinol binding site, and a *c*-type cytochrome. The cytochrome *c* subunit is least conserved among bacteria and usually is of a mono- or di-heme, sometimes also a tetraheme, type (Ilbert and Bonnefoy, 2012). *N. gracilis* has one operon that shows moderate similarity to complex III (Table S1 in Supplementary Material). The three proteins encoded in this cytochrome *bc*-like operon show highest similarity to the cyanobacterial cytochrome *b_6_f* rather than to the *bc_1_* complex. They consist of a Fe-S subunit next to a split cytochrome *b* subunit. This fission of the cytochrome *b* into a cytochrome *b_6_* and a so-called subunit IV is observed in many non-phototrophic bacteria that have this type of complex III (Sone et al., 1995; Schütz et al., 2000). Notably, *Nitrospina* carries elsewhere in the genome a second copy of the cytochrome *b* subunit that is not split, but also appears to be of the *b/b_6_*-type (Table S1 in Supplementary Material). The only putative cytochrome *c* subunit encoded next to the cytochrome *bc*-like operon is an atypical octaheme cytochrome *c*. Interestingly, the exactly same operon structure including a similar octaheme protein is conserved in the genome of the anammox organism “*Candidatus* Kuenenia stuttgartiensis” (de Almeida et al., 2011) and in “*Candidatus* Methylomirabilis oxyfera,” a noteworthy bacterium capable of nitrite-driven anaerobic methane oxidation (Ettwig et al., 2010).

The genome of *N. gracilis* contains the genes for all 14 subunits of NADH:quinone oxidoreductase (NUO; complex I) needed for the forward transport of electrons from NADH to quinone (Figure 3; Table S1 in Supplementary Material). All Fe-S clusters required for electron channeling between NADH and quinone are preserved, as are the NADH and flavin mononucleotide (FMN)-binding patterns within subunit NuoF. The *nuoA-M* genes are clustered in one operon on the lagging strand, whereas *nuoN* is located on the opposite strand five genes downstream of *nuoA*. Besides the canonical NADH:quinone oxidoreductase (NUO-1), a second copy is present, but the genes coding for this NUO-2 are distributed across the genome (Table S1 in Supplementary Material). While *nuoB2* and *nuoF2* are not organized in an operon structure, the other genes of NUO-2 form two clusters consisting of *nuoACHIJKLMN* and *nuoEGD*, respectively. Intriguingly, this complex shows substantial differences to NUO-1 in the binding patterns for cofactors in the catalytic subunits. NADH in canonical NUOs (including NUO-1 of *N. gracilis*) binds to NuoF, where two electrons are transferred onto the primary electron acceptor FMN that serves as a two-to-one-electron converter (Brandt, 2006). Consecutively, FMN feeds single electrons into a chain of seven conserved Fe-S clusters distributed throughout subunits NuoE, G, I, and B in the hydrophilic arm of the complex, at the end of which quinone reduction occurs (Sazanov and Hinchliffe, 2006). The *N*. *gracilis* NuoF2 lacks the FMN binding site, but contains one [2Fe-2S] and two [4Fe-4S] ferredoxin-like clusters in addition to the Fe-S cluster N3 and the NADH binding domain. Similarly, NuoG2 has the characteristic cysteine binding patterns for Fe-S clusters N1b, N4, N5, and N7, along with one additional [4Fe-4S] cluster. Without the FMN cofactor a function as NUO is unlikely, but a possible alternative role for NUO-2 might be in the reverse electron transport from quinol to low-potential ferredoxin. Two enzymes of the rTCA cycle (see below) belong to the 2-oxoacid:ferredoxin oxidoreductase family and require reduced low-potential ferredoxin as electron donor for the reductive carboxylation of their substrates. Common mechanisms to reduce low-potential ferredoxin with electrons from NADH (Rnf complex; Biegel and Müller, 2010) or H_2_ (Ech complex; Welte et al., 2010) employ membrane-integral complexes that use the proton motive force for reverse electron transport to ferredoxin ($E^{0^{\prime }}\;=\;-500$ to -420 mV). These complexes evolved from a NUO-like ancestor (Buckel and Thauer, 2013). *Nitrospina* lacks these known pathways, but the NUO-2 complex is an interesting candidate to perform ferredoxin reduction with quinol as electron donor. Due to the lack of the FMN binding site, the NuoF2 subunit cannot interact with a two-electron carrier such as NAD+/NADH. However, as NADH and ferredoxin binding sites display homology in some proteins (Hanke et al., 2011), the predicted NADH binding site of NUO-2 might interact with the single-electron carrier ferredoxin. Powered by proton motive force and with the help of the four additional Fe-S clusters in NuoG2 and NuoF2, two of which are of the bacterial [4Fe-4S] ferredoxin type, it might be possible to elevate electrons from the quinol pool not only to the redox potential of NADH, but further to the potential of ferredoxin. Indeed, the ferredoxin-like [4Fe-4S] clusters 6a and 6b of NuoI display a negative shift of their redox midpoint potential of <600 mV when expressed heterologously without incorporation into the NUO complex (Yano et al., 1999), and the NuoI homolog in Ech is responsible for ferredoxin reduction (Forzi et al., 2005). The extra [4Fe-4S] clusters in NuoF2 could thus be sufficiently electronegative to interact with ferredoxin. Interestingly, in *Ca*. K. stuttgartiensis a highly similar complex is present that also contains all additional Fe-S clusters in the NuoGF subunits and apparently lacks a FMN binding site. The organization of all corresponding genes in one operon in *Kuenenia* strongly suggests that their products are subunits of the same enzyme complex. The reduction of ferredoxin by reverse electron transport is important also in *Kuenenia*, which employs the acetyl-coenzyme A pathway for CO_2_ fixation (Strous et al., 2006). The NUO-2 complex of *Kuenenia* has been tentatively annotated as formate:quinone oxidoreductase (Strous et al., 2006), but biochemical data endorsing this function is lacking. Future experimental work may reveal whether the NUO-2 complex indeed is a novel mechanism for reverse electron transport spanning the large difference in redox potential between quinol and ferredoxin.

Complex II, the succinate:quinone oxidoreductase (SQR), links the TCA cycle to the quinone pool. This complex can work in both directions. It catalyzes either the oxidation of succinate in the course of the oxidative TCA cycle and oxidative phosphorylation, or the reduction of fumarate to channel electrons into the rTCA cycle (Cecchini et al., 2002). SQRs are classified in five types (A–E; Lemos et al., 2002) and consist of three (type B and E) or four (type A, C, and D) subunits. Many organisms encode a separate succinate dehydrogenase (SDH) and fumarate reductase (FRD), which are expressed differentially under oxic and anoxic conditions, but can also replace each other functionally (Maklashina et al., 1998). SDH and FRD in the same organism usually are similar in sequence and subunit composition, or only one enzyme is present. In contrast, *N*. *gracilis* contains two highly different versions of SQRs. One enzyme is of type B that is found also in bacilli and *Epsilonproteobacteria*, whereas the other one belongs to type E found mainly in *Archaea* (Lemos et al., 2002). Both types consist of three subunits (Table S1 in Supplementary Material). The type B SQR-1 of *N. gracilis* consists of a FAD-binding flavoprotein, a Fe-S protein containing all conserved cysteine residues for binding one [2Fe-2S], one [4Fe-4S], and one [3Fe-4S] cluster, and a diheme cytochrome *b* membrane subunit with five predicted transmembrane helices (Lancaster and Simon, 2002). This SQR type is a good candidate for a fumarate reductase, because it interacts with menaquinone (MQ) rather than ubiquinone (Schirawski and Unden, 1998) and MQ has a lower redox potential ($E^{0^{\prime }}=-74\text{mV}$) than the succinate/fumarate couple ($E^{0^{\prime }}=+30\text{mV}$). In *Bacillus subtilis* fumarate reduction with menaquinol catalyzed by a type B SQR is electrogenic, so that the reaction contributes to the proton gradient over the cytoplasmic membrane (Schnorpfeil et al., 2001; Lancaster et al., 2008). However, the quinone types present in *Nitrospina* are not known yet. The type E SQR-2 has some fundamental differences to the type B SQR-1 in its subunit composition and predicted binding sites for prosthetic groups. The large subunit also is a FAD-binding flavoprotein, but it has only low similarity to the corresponding subunit of SQR-1. The Fe-S subunit contains a second [4Fe-4S] cluster instead of the [3Fe-4S] cluster (Gomes et al., 1999), resulting in a slightly lower redox potential of the binuclear center (Lemos et al., 2001). Further, type E SQRs contain an unusual membrane subunit (SdhE) that is unrelated to the membrane anchors of the SQR types A–D. The respective gene is split in the *N. gracilis* genome sequence, but this may be caused by an uncorrected sequencing error. SdhE does not bind heme *b*, has only one small putative membrane-spanning segment at the C-terminus, and contains two tandem repeats of the conserved cysteine motif CX_31_CCGX_34_CX_2_C (Janssen et al., 1997). These motifs bind an additional high-potential [2Fe-2S] cluster that functionally replaces the missing heme *b* (Iwasaki et al., 2002). Despite these differences, SdhE is essential for membrane attachment of the SQR complex, possibly mediated by amphipathic helices, and (mena-)quinone interaction (Lemos et al., 2002). Although type E SQRs can work reversibly, they appear to preferentially catalyze succinate oxidation in most organisms carrying these enzymes (Gomes et al., 1999). The slightly lower redox potential of the active center allows this SQR to efficiently donate electrons to quinone or directly to Rieske-type Fe-S proteins as found in complex III (Janssen et al., 1997). Hence, the SQR-2 of *N. gracilis* is the more likely candidate than SQR-1 for a SDH that is active during the degradation of carbon storage compounds.

Interestingly, *N. gracilis* contains genes for a cytoplasmic NiFe-hydrogenase (Figure 3; Table S1 in Supplementary Material). These genes are organized in a single operon and encode the four subunits of the holoenzyme and six accessory proteins involved in hydrogenase assembly and maturation. Sequence analysis revealed binding sites for nickel in the α subunit and for one and three [4Fe-4S] clusters in the β and δ subunit, respectively. The γ subunit contains predicted binding sites for one [2Fe-2S] cluster, FAD, and NAD(P). Characteristic amino acid patterns in the large subunit clearly classify the enzyme as a type 3b bidirectional (NADP) hydrogenase (Vignais and Billoud, 2007). Members of this group catalyze the reversible oxidation of H_2_ with NAD(P)^+^ (Silva et al., 2000). Furthermore, most type 3b hydrogenases can reduce elemental sulfur (S^0^) or polysulfide to H_2_S. Since they display higher affinities for polysulfides than for H^+^, they are also referred to as sulfhydrogenases (Ma et al., 2000). The physiological role of this enzyme in *N. gracilis* is unclear, but three functions appear to be possible: (i) When present, H_2_ may be oxidized to provide reduced NAD(P)H for biosynthetic processes and energy generation. (ii) Polysulfide (or H^+^) can be used as terminal electron acceptor during fermentation of intracellular glycogen deposits in absence of more favorable electron acceptors. (iii) Polysulfide (or S^0^) can be reduced to H_2_S for sulfur assimilation during protein biosynthesis if neither sulfate nor sulfite is available. None of these reactions has been reported for *N. gracilis* so far and all three possibilities seem unlikely in the aerobic marine habitat. However, the hydrogenase may be advantageous under hypoxic or even anoxic conditions (see also below).

*N. gracilis* encodes a periplasmic sulfite:cytochrome *c* oxidoreductase that may couple sulfite oxidation to sulfate with the reduction of two cytochromes (Figure 3; Table S1 in Supplementary Material). In eukaryotes this reaction mainly serves as a detoxification mechanism for sulfite formed during the degradation of sulfur-containing amino acids, but it can be used for energy conservation by sulfur-oxidizing prokaryotes (Kappler et al., 2000). Since *N. gracilis* also has all genes needed for sulfate assimilation *via* 3′-phosphoadenylyl sulfate and the subsequent reduction to sulfide, the sulfite:cytochrome *c* oxidoreductase is probably not involved in sulfur assimilation. Hence, *N. gracilis* might be able to use sulfite as alternative energy and/or electron source, but physiological data showing this metabolic capacity are lacking.

### CO_2_ fixation and carbon metabolism

According to cultivation-based experiments, *N. gracilis* is an obligate autotroph that meets all carbon demands by CO_2_ fixation (Watson and Waterbury, 1971). Surprisingly, *Nitrospina* employs the rTCA cycle for this task. This CO_2_ fixation pathway is unexpected in an aerobic organism, because two of its key enzymes belong to the 2-oxoacid oxidoreductase (OR) family whose members normally are highly oxygen-sensitive (Berg, 2011). However, *Hydrogenobacter thermophilus* possesses unusual five-subunit types of 2-oxoglutarate:ferredoxin (OGOR) and pyruvate:ferredoxin oxidoreductase (POR) that are functional under oxic conditions (Yoon et al., 1996, 1997). Moreover, the aerobic nitrite oxidizer *Ca*. N. defluvii also utilizes this pathway using such five-subunit OGOR and POR (Lücker et al., 2010). *N. gracilis* encodes a five-subunit POR with high identity of the subunits (60–76%) to the *Nitrospira* holoenzyme. The OGOR of *N. gracilis* lacks the δ subunit, but the remaining subunits also share high identities with their homologs in *Nitrospira* (53–68%; Table S1 in Supplementary Material). The δ subunit was required for heterologous expression of the *H. thermophilus* holoenzyme in *E. coli*, but it does not contain any known binding motifs for prosthetic groups (Ikeda et al., 2006). A function of this subunit in the expression or assembly of the active OGOR complex might be fulfilled by the δ subunit of POR or by a yet unidentified chaperone. The reduced ferredoxins required by the two ORs could either be provided by the alternative NUO-2 complex (see above) or by a complex involving electron transfer flavoproteins (ETFs). *Clostridium kluyveri* can couple the reduction of ferredoxin with NADH to crotonyl-CoA reduction, similar to the reaction in the ETF:quinone reductase in mammals (Herrmann et al., 2008). In this electron bifurcation reaction, the energy derived from the electron transfer from NADH to crotonyl-CoA is used to simultaneously catalyze the unfavorable reduction of a [4Fe-4S] ferredoxin (Buckel and Thauer, 2013). The two FAD-containing ETFs as well as a putative FAD-containing acyl-CoA dehydrogenase necessary are encoded in the *N. gracilis* genome (Table S1 in Supplementary Material), but it appears counterintuitive that *Nitrospina* should rely on the fatty acid degradation pathway for the reduction of ferredoxin during autotrophic growth. Alternatively, the ETF proteins might interact with an unidentified flavin-containing ETF:quinone reductase, that couples the ferredoxin reduction by NADH to the reduction of quinone.

Besides OGOR and POR, the presence of ATP citrate lyase (ACL) is generally considered to be a prerequisite of the rTCA cycle (Hügler et al., 2007). The *N. gracilis* genome contains two CDS with very low similarities to the large and small subunits of known ACLs (Table S1 in Supplementary Material). They contain the characteristic succinyl-CoA synthase-like NAD(P)- and CoA-binding domains and the ATP-grasp fold (Kanao et al., 2001) in the large and small subunit, respectively. The alternative pathway of ATP cleavage in *Aquificaceae* and *Leptospirillum*, which involves citryl-CoA synthase and citryl-CoA lyase (Hügler et al., 2007; Levican et al., 2008), could not be identified in *N. gracilis*. Therefore, we assume that the predicted ACL is functional despite its low similarity to validated ACLs from other organisms.

Each of the other enzymes involved in the reductive and/or oxidative TCA cycles (Berg, 2011) has one homolog in the *N. gracilis* genome (Table S1 in Supplementary Material). There are, however, notable differences to the typical enzyme set for the anaplerotic reactions replenishing the oxaloacetate pool: usually, oxaloacetate is formed from pyruvate either in one single step by pyruvate carboxylase or *via* phosphoenolpyruvate (PEP) by the enzymes pyruvate, water dikinase, and PEP carboxylase. Intriguingly, *N. gracilis* lacks these enzymes, but alternatives for these routes are present. Firstly, instead of pyruvate carboxylase *Nitrospina* appears to employ a membrane-bound oxaloacetate decarboxylase. This enzyme complex usually performs the oxidative decarboxylation of oxaloacetate and directly uses the released energy for the extrusion of sodium ions across the membrane. In the presence of a sodium gradient, however, the reaction can be reversed to carboxylate pyruvate (Dimroth and Hilpert, 1984). Such dependency on a sodium gradient to drive this reaction might also explain why *Nitrospina* is restricted to marine habitat types. Secondly, the two-step route via PEP can be mediated by the enzymes pyruvate, phosphate dikinase, and the ATP-utilizing PEP carboxykinase. Pyruvate, phosphate dikinase replaces pyruvate, water dikinase in many phototrophic organisms, but usually functions in ATP synthesis from PEP in chemoautotrophic bacteria (Lim et al., 2007). The second enzyme, PEP carboxykinase, is mainly involved in gluconeogenesis by replenishing PEP from oxaloacetate, but can also catalyze the carboxylation of PEP (Zamboni et al., 2004).

Observation by electron microscopy of *N. gracilis* cells revealed glycogen deposits as intracellular storage compounds (Watson and Waterbury, 1971). Accordingly, automatic genome annotation predicted all genes required for gluconeogenesis and glycogen formation (Figure 3; Table S1 in Supplementary Material). The stored glycogen can be catabolized and used as energy source *via* the Embden–Meyerhof–Parnas pathway and the oxidative (oTCA). Organotrophic growth of *N. gracilis* was not observed previously (Watson and Waterbury, 1971). Consistently, no transport systems for the import of organic substrates were identified in the genome except one, merely putative, ABC-type sugar transporter of unknown specificity.

### Resistance and defense

Although *N. gracilis* is an aerobic organism, it lacks classical defense mechanisms against reactive oxygen species (ROS) usually found in aerobic bacteria. No homolog of superoxide dismutase (SOD) or reductase (SOR) could be identified in the genome, and catalase appears to be absent, too. Hydrogen peroxide might be degraded by a cytochrome *c* peroxidase and peroxiredoxins, possibly assisted by the thioredoxin and glutaredoxin systems, which all are encoded in the genome. The protection mechanism against superoxide radicals is more puzzling. *Ca*. N. defluvii also lacks SOD and catalase, and the presence of a manganese import system has led to speculations that some resistance of this organism against ROS may be conferred by manganese (Lücker et al., 2010). Although the *N. gracilis* genome contains no known specific manganese import system, protection from ROS in the periplasm might be mediated by a periplasmic multicopper protein that is highly conserved in the *Ca*. N. defluvii genome, too. This putative multicopper oxidase shows similarity to an enzyme of *Bacillus* sp. strain SG-1 that is capable of manganese oxidation to MnO_2_ (van Waasbergen et al., 1996). Since multicopper oxidases are one-electron oxidants, the oxidation of Mn(II) to Mn(IV) proceeds via a Mn(III) intermediate (Spiro et al., 2009). Mn(III) can be stabilized in the cell by binding to siderophores (Parker et al., 2004) or pyrophosphate (Tebo et al., 2004), and Mn(III) pyrophosphates contribute up to 100% of the total dissolved manganese pool in marine suboxic zones (Trouwborst et al., 2006). Mn(III) can oxidize ${\text{O}}_{2^{-}}^{∙}$ to harmless O_2_, becoming reduced back to Mn(II) (Barnese et al., 2008; Batinic-Haberle et al., 2011). Interestingly, *N. gracilis* also has genes for siderophores that may function in Mn(III) stabilization. One could speculate that siderophores loaded with Mn(III) may also be transported into the cell, extending ROS protection from the periplasmic space into the cytoplasm. Here, pyrophosphate for further Mn(III) stabilization could be provided by inorganic phosphatase. Thus, Mn(III) produced by the multicopper oxidase could be stabilized and may contribute to ROS protection. Aside from manganese, polyamines can confer protection against oxidative stress and scavenge free radicals (Chattopadhyay et al., 2003; Tkachenko et al., 2012). As the *N. gracilis* genome contains the genes needed for producing the polyamines putrescine and spermidine, the organism might also employ this ROS defense mechanism.

*N. gracilis* appears to be well protected against a plethora of toxic compounds (Table S1 in Supplementary Material). Arsenate reductase (Ji and Silver, 1992) and mercuric reductase (Laddaga et al., 1987) were identified in the genome. Thiosulfate sulfurtransferase and cyanate hydratase convert thiocyanate to cyanate and then to ammonia and CO_2_ for cyanate detoxification (Kamennaya et al., 2008). This mechanism may also allow *N. gracilis* to use (thio)cyanate as source of ammonium for assimilation. A range of transporters for monovalent copper, diverse mono- and divalent cations, acriflavin, as well as multidrug export systems complete the diverse defense mechanisms of *N. gracilis*. Multiple Na^+^ export systems, including an putative eight subunit Mnh-type secondary Na^+^/H^+^antiporter (Swartz et al., 2007), likely contribute to salt tolerance necessary for growth in the marine environment.

## Discussion

### Phylogeny and diversity of *Nitrospina*

As each gene is shaped by a unique set of functional constraints through evolution, phylogenetic trees based on different single genes, which are calculated for the same set of organisms, may show conflicting topologies (Rokas et al., 2003). Therefore, comparative phylogenetic analyses using different individual marker genes often suffer from incongruent trees that hamper firm conclusions on the affiliation of an organism or even a whole lineage. This problem can be ameliorated by using concatenated datasets of multiple marker genes or proteins, as analyses based on such datasets are not biased toward the evolutionary history of a single marker (Rokas et al., 2003; Strous et al., 2006). This approach and the now available genome sequence of *N. gracilis* allowed us to assess the phylogenetic affiliation of *Nitrospina* with an unprecedented high degree of confidence. Contrasting previous phylogenies, which were based on 16S rRNA gene sequences only (Teske et al., 1994), our analyses strongly suggest that *N*. *gracilis* does not belong to the *Deltaproteobacteria* but represents a distinct, deep-branching line of descent within the *Bacteria*. This conclusion gains further support from analyses of conserved insertions and deletions in highly conserved genes (Gupta and Griffiths, 2002) and of gene transposition events (Kunisawa, 2006), which are not affected by the quality of sequence alignments and other possible biases of tree calculation methods. For the novel phylum we therefore propose the tentative name *Nitrospinae* phyl. nov. and the reclassification of the family *Nitrospinaceae* (Garrity et al., 2005) in the *Nitrospinia* class. nov., and *Nitrospinales* ord. nov., with *N. gracilis* as the type species. The species description remains as published by Watson and Waterbury (1971). However, the exact position of the *Nitrospinae* in the bacterial tree could not be resolved unambiguously. Some trees suggested a position between the *Proteobacteria* and a group consisting of *Nitrospirae* and *Acidobacteria*, whereas others indicated a superphylum comprising the *Nitrospirae*, *Acidobacteria*, and *Nitrospinae* (Figure 1). Ambiguous branching patterns of underrepresented lineages in phylogenetic trees can sometimes be resolved by adding more reference sequences related to these lineages (Peplies et al., 2008). However, this is currently not possible for *Nitrospina* since no closely related organisms are known.

### Evolutionary history of nitrite oxidation

**Figure 5:**
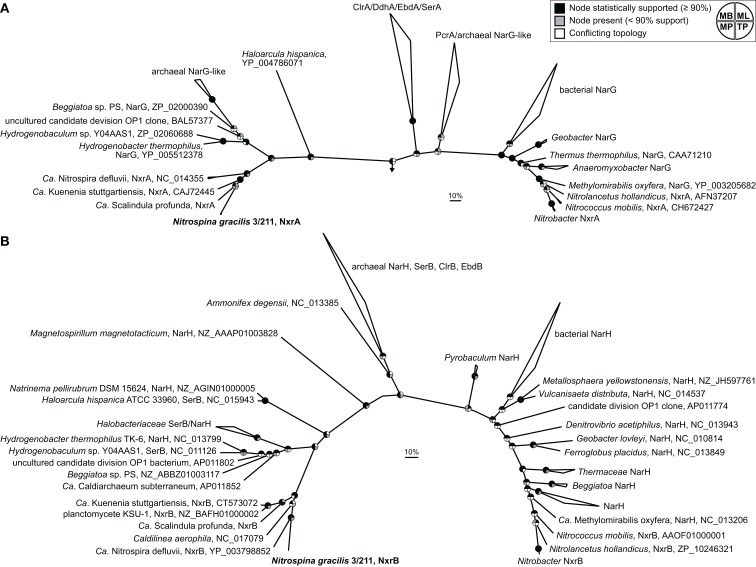
**Phylogeny of *N. gracilis* 3/211 NXR (boldface) and related enzymes**. Both trees show selected enzymes from the DMSO reductase type II family. Names of validated enzymes are indicated (Clr, chlorate reductase; Ddh, dimethylsulfide dehydrogenase; Ebd, ethylbenzene dehydrogenase; Nar, Nitrate reductase; Nxr, nitrite oxidoreductase; Pcr, perchlorate reductase; Ser, selenate reductase). More distantly related molybdoenzymes were used as outgroup. The scale bar represents 10% estimated sequence divergence. Pie charts indicate statistical support of nodes based on Bayesian inference, bootstrap analysis, or treepuzzle support. MB, Bayesian inference; ML, maximum-likelihood (RAxML); MP, maximum-parsimony (ProtPars); TP, maximum-likelihood (TreePuzzle). **(A)** Bayesian interference tree (SD = 0.006924) of the large (α) subunit. In total, 1,475 alignment positions were considered. **(B)** Bayesian interference tree (SD = 0.015471) of the small (β) subunit, obtained by using 532 alignment positions.

Two main groups of NXR are known which can be distinguished based on their subcellular localization and phylogenetic affiliation within the type II DMSO reductase-family molybdopterin cofactor-binding enzymes. In *Nitrobacter*, *Nitrococcus*, and *Nitrolancetus* NXR faces the cytoplasmic side of the cytoplasmic membrane. This type of NXR is closely related to the respiratory nitrate reductase (NAR) of *E. coli* and many denitrifying bacteria (Lücker et al., 2010; Sorokin et al., 2012). Furthermore, the proteobacterial NOB *Nitrobacter* and *Nitrococcus* contain stacks of intracytoplasmic membranes (ICM), which are densely packed with NXR complexes also facing the cytoplasm (Spieck and Bock, 2005a). Since these ICM resemble those of related phototrophic proteobacteria (purple bacteria), Teske et al. (1994) hypothesized that *Nitrobacter* and *Nitrococcus* evolved from phototrophic ancestors where nitrite was used as electron donor for anoxygenic photosynthesis. Indeed, phototrophic nitrite oxidizers from the genera *Rhodopseudomonas* and *Thiocapsa*, which are closely related to *Nitrobacter* and *Nitrococcus*, were discovered recently (Schott et al., 2010). In contrast, *Nitrospira* lack ICM. Their NXR is also attached to the cytoplasmic membrane, but it is oriented toward the periplasm (Spieck et al., 1998; Lücker et al., 2010). Previously, we showed that this periplasmically oriented NXR is phylogenetically distinct from the cytoplasmically oriented enzyme and clusters together with the NXR of the anammox organism *Ca*. K. stuttgartiensis (Lücker et al., 2010). Anammox bacteria oxidize nitrite not for energy generation but to replenish the respiratory chain with electrons needed for CO_2_ fixation (Jetten et al., 2009). Interestingly, the different subcellular orientation and phylogenetic separation of the two NXR types mirror differences in the nitrite requirements of the respective NOB, which were determined experimentally (Schramm et al., 1999; Bartosch et al., 2002; Freitag et al., 2005; Sorokin et al., 2012). All known NOB with the periplasmic NXR achieve growth with lower nitrite concentrations than NOB with the cytoplasmic enzyme (Sorokin et al., 2012). A likely explanation is the higher efficiency of periplasmic NXR, which conserves more energy per oxidized nitrite. Since the additional O atom in nitrate stems from water, two protons are released in the periplasmic space if nitrite is oxidized by a periplasmic NXR. An additional two protons are consumed in the cytoplasm during O_2_ reduction to water by the terminal oxidase. Thus, the activity of a periplasmic NXR directly contributes to the proton motive force. This is not the case with a cytoplasmically oriented NXR, where the protons are released and consumed at the same side of the membrane (Lücker et al., 2010). *N. gracilis* lacks ICM and is adapted to low nitrite concentrations (Watson and Waterbury, 1971). Consistently, its two copies of the NxrA subunit are highly similar to the periplasmic NxrA of *Ca*. N. defluvii (54–55% identity). Surprisingly, however, their sequence identity to the NxrA of *Ca*. K. stuttgartiensis is even higher (62%), indicating a closer evolutionary relationship between the NXRs of *Nitrospina* and anammox bacteria. Indeed, phylogenetic analysis showed that the NxrA of *Nitrospina* clusters together with the marine “*Ca*. Scalindula profunda” within the anammox group, whereas *Nitrospira* branches off at the ancestral node of the NXR branch (Figure 5A). This branching pattern implies that the genes coding for the periplasmic NXR were horizontally transferred from an ancestor of *Nitrospira* to an ancestral anammox organism (or vice versa) and from a (putatively marine) anammox bacterium to *Nitrospina*. Vertical inheritance can be excluded due to the large phylogenetic distance between these organisms at the inter-phylum level. The first HGT between ancestral *Nitrospira* and anammox requires the coexistence of these organisms in an anoxic or hypoxic environment, which was already proposed based on the first analysis of a *Nitrospira* genome (Lücker et al., 2010). Since *Nitrospina*-like bacteria often co-occur with anammox organisms in low-oxygen marine environments (Füssel et al., 2012), the second HGT from anammox to *Nitrospina* appears possible as well. The use of the rTCA cycle for carbon fixation also is fully consistent with a microaerophilic or even anaerobic evolutionary origin of *Nitrospina*. The periplasmic NXR might originally have evolved in ancestral *Nitrospira* or anammox bacteria, or it was received by one of these lineages from an unknown donor. As the closest known branch contains bacterial and archaeal nitrate reductases (Figure 5A), one may speculate that such a donor was a nitrate-reducing or nitrite-oxidizing bacterium or archaeon. In this context it appears striking that the reaction of recent periplasmic NXR is reversible and the enzyme still catalyzes both nitrite oxidation and nitrate reduction (Ehrich et al., 1995).

In phylogenetic trees based on the smaller and highly conserved NxrB subunit, *N. gracilis* and *Nitrospira* cluster together and the anammox lineage branches off first (Figure 5B). This topology is unclear with respect to the aforementioned order of HGT events, because the common ancestral node may represent a member of the anammox lineage, *Nitrospirae*, *Nitrospinae*, or an unknown donor. Additional NxrB sequences from each lineage, once available, might disambiguate the branching order. However, the clustering of the NxrB of *Nitrospira* and *Nitrospina* may also reflect similar functions in the respiratory chain. NxrB is more downstream than NxrA and channels electrons to the gamma subunit, NxrC. In both aerobic NOB, the electrons from nitrite are shuttled either toward a terminal oxidase or into a reverse electron transport chain. This bifurcated electron flow from nitrite may require different forms of NxrC, which interact with the respective downstream acceptors. Consistently, the genomes of both *Ca*. N. defluvii and *N. gracilis* contain several different NxrC-like CDS (Lücker et al., 2010 and this study). NxrB must then interact with different types of NxrC. This flexibility may require adaptations in the sequence of NxrB not found in anammox bacteria, where nitrite oxidation is always followed by reverse electron flow. Alternative explanations for the differences between the NxrA and NxrB trees include the possibility that these NXR subunits do not share the same evolutionary history but were subject to different HGT events. Interestingly, a member of the *Chloroflexi*, *Caldilinea aerophila*, also encodes an NxrB-like protein that branches between *Nitrospina*/*Nitrospira* and the anammox group (Figure 5B). A closer inspection of its publicly available genome sequence revealed that *C. aerophila* also carries genes for the TorD-like chaperone and NxrC, but the catalytic NxrA is lacking. Thus, the functions of its NXR-like proteins remain to be investigated.

**Figure 6:**
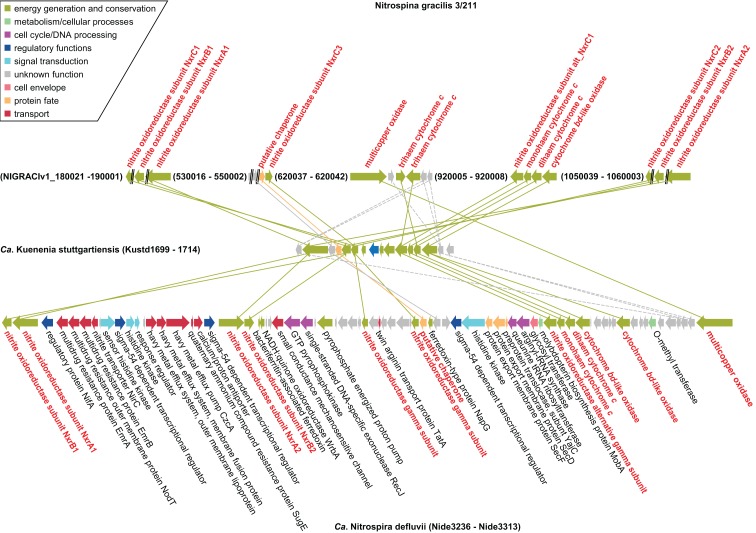
**Schematic illustration of the genomic regions in *N. gracilis* 3/211, *Ca*. K. stuttgartiensis, and *Ca*. N. defluvii that contain genes coding for NXR, chaperones, electron carriers, cytochrome *bd*-like oxidases, multicopper oxidases, and conserved proteins of unknown function**. Solid lines indicate proteins that are closest homologs based on protein phylogeny. Dashed lines connect homologous proteins that are not the closest relatives in the respective phylogenetic trees. Proteins and connecting lines are color-coded according to functional classes. CDS and intergenic regions are drawn to scale.

Phylogenomics (Frickey and Lupas, 2004), further illuminated the extent of HGT between *Nitrospina*, *Nitrospira*, and the anammox lineage (represented by *Kuenenia*; Figure 2). A set of genes is highly conserved among all three organisms (Figure 6). In *Ca*. K. stuttgartiensis these genes form one cluster that codes for the NXR subunits and other proteins that may form a membrane-bound complex involved in nitrite oxidation and electron transfer into the respiratory chain (de Almeida et al., 2011). Exactly these genes are conserved between *Kuenenia* and *Nitrospira* (Lücker et al., 2010), and their conservation in *Nitrospina* strongly supports their importance for nitrite oxidation in these bacteria. Besides the NXR α, β, and γ subunits, these genes encode an alternative NxrC subunit, the TorD-like chaperone, one mono- and one diheme cytochrome *c*, one putative zinc finger- and two cupredoxin-like copper-binding proteins, and one conspicuous cytochrome *bd*-like oxidase. The two cytochromes *c* likely are involved in electron transport from NXR to the terminal cytochrome *c* oxidase for energy generation and to the cytochrome *bc* complex for reverse electron transport. Alternatively, the interaction with the terminal oxidase or the *bc* complex might involve copper instead of heme-based electron carriers. This would explain the presence of genes coding for cupredoxin-like proteins in the cluster (see also de Almeida et al., 2011). The function of the *bd*-like oxidase is more enigmatic. The predicted protein contains 14 transmembrane helices and shows partial similarity to subunit I of canonical cytochrome *bd* terminal oxidases. In comparative analyses, the N-terminal parts of the canonical *bd* and *bd*-like oxidases align well and the ligands for heme *b*-binding (Borisov et al., 2011) are conserved. In place of the periplasmically oriented Q-loop found in canonical *bd* oxidases, however, the *bd*-like oxidases have three predicted membrane helices, and all residues responsible for quinol binding in the canonical enzyme (Borisov et al., 2011) are lacking. Thus, unless the *bd*-like oxidases have evolved an alternative quinol binding site, a role comparable to canonical cytochrome *bd* terminal oxidases is unlikely. However, if the enzymes can interact with quinone they might function like an alternative complex III and enable reverse electron transport from nitrite to the quinone pool, either through direct interaction with NXR, or mediated by the copper- or heme-based electron carriers also encoded in the gene cluster. Interestingly, the gene cluster also contains the gene for the aforementioned multicopper oxidase that might be involved in ROS defense in *Nitrospina* and *Nitrospira* (Figure 6). The lack of this gene in *Ca*. K. stuttgartiensis would be in line with a function in ROS defense, which would be largely obsolete in strictly anaerobic anammox bacteria.

## Conclusion

*N. gracilis* is the most abundant and widespread nitrite oxidizer in marine systems and is of central importance to the oceanic nitrogen cycle. The extended phylogenetic analyses performed in this study have shown that *N. gracilis* represents a novel bacterial phylum. Intriguingly, *N. gracilis* extends an evolutionary model of nitrite oxidation that is based on lateral gene transfer across phylum boundaries, and it strengthens the previously proposed evolutionary link between anammox bacteria and NOB with a periplasmic NXR (Lücker et al., 2010; Sorokin et al., 2012). Like with *Nitrospira*, the use of an oxygen-sensitive CO_2_ fixation pathway and the lack of canonical ROS defense mechanisms suggest an evolutionary origin of *Nitrospina* from microaerophilic or even anaerobic ancestors. Consistently, *Nitrospina*-like organisms are frequently encountered in hypoxic marine habitats. However, nitrite accumulation in the truly anoxic core regions of OMZs (Thamdrup et al., 2012) indicates that the observed nitrite oxidation across OMZs, and also in marine sediments, still requires oxygen and probably is caused by the activity of microaerophilic NOB including *Nitrospina*.

## Conflict of Interest Statement

The authors declare that the research was conducted in the absence of any commercial or financial relationships that could be construed as a potential conflict of interest.

## Supplementary Material

The Supplementary Material for this article can be found online at http://www.frontiersin.org/Evolutionary_and_Genomic_Microbiology/10.3389/fmicb.2013.00027/abstract

Supplementary Table S1 ***Nitrospina gracilis* strain 3/211 proteins with predicted functions in key metabolic pathways**.

## Appendix

**Table A1:** **Proteins used for phylogeny**.

Gene	Protein	Identifier	Alignment positions
rplM	Ribosomal protein L1	NIGRACIv1_180012	244
rplB	Ribosomal protein L2	NIGRACIv1_280027	305
rplC	Ribosomal protein L3	NIGRACIv1_280024	288
rplD	Ribosomal protein L4	NIGRACIv1_280025	233
rplE	Ribosomal protein L5	NIGRACIv1_280036	181
rplF	Ribosomal protein L6	NIGRACIv1_280039	165
rplL	Ribosomal protein L7/12	NIGRACIv1_280016	137
rplI	Ribosomal protein L9	NIGRACIv1_620016	164
rplJ	Ribosomal protein L10	NIGRACIv1_280015	139
rplK	Ribosomal protein L11	NIGRACIv1_280013	156
rplM	Ribosomal protein L13	NIGRACIv1_180012	164
rplN	Ribosomal protein L14	NIGRACIv1_280034	163
rplO	Ribosomal protein L15	NIGRACIv1_280042	230
rplP	Ribosomal protein L16	NIGRACIv1_280031	146
rplQ	Ribosomal protein L17	NIGRACIv1_280051	150
rplR	Ribosomal protein L18	NIGRACIv1_280040	126
rplS	Ribosomal protein L19	NIGRACIv1_380018	119
rplT	Ribosomal protein L20	NIGRACIv1_220025	114
rplU	Ribosomal protein L21	NIGRACIv1_1040055	117
rplV	Ribosomal protein L22	NIGRACIv1_280029	111
rplW	Ribosomal protein L23	NIGRACIv1_280026	128
rplX	Ribosomal protein L24	NIGRACIv1_280035	165
rpmA	Ribosomal protein L27	NIGRACIv1_1040054	85
rpmB	Ribosomal protein L28	NIGRACIv1_500015	47
rpmC	Ribosomal protein L29	NIGRACIv1_280032	44
rpmF	Ribosomal protein L32	NIGRACIv1_740027	62
rpmI	Ribosomal protein L35	NIGRACIv1_220024	46
rpsB	Ribosomal protein S2	NIGRACIv1_250010	224
rpsC	Ribosomal protein S3	NIGRACIv1_280030	257
rpsD	Ribosomal protein S4	NIGRACIv1_280049	248
rpsE	Ribosomal protein S5	NIGRACIv1_280041	149
rpsF	Ribosomal protein S6	NIGRACIv1_620012	116
rpsG	Ribosomal protein S7	NIGRACIv1_280020	212
rpsH	Ribosomal protein S8	NIGRACIv1_280038	170
rpsI	Ribosomal protein S9	NIGRACIv1_180011	154
rpsJ	Ribosomal protein S10	NIGRACIv1_280023	98
rpsK	Ribosomal protein S11	NIGRACIv1_280048	114
rpsL	Ribosomal protein S12	NIGRACIv1_280019	151
rpsM	Ribosomal protein S13	NIGRACIv1_280047	123
rpsO	Ribosomal protein S15	NIGRACIv1_950034	84
rpsP	Ribosomal protein S16	NIGRACIv1_380023	66
rpsQ	Ribosomal protein S17	NIGRACIv1_280033	71
rpsR	Ribosomal protein S18	NIGRACIv1_620014	76
rpsS	Ribosomal protein S19	NIGRACIv1_280028	88
rpsT	Ribosomal protein S20	NIGRACIv1_270042	110
rpoA	RNA polymerase, alpha subunit	NIGRACIv1_280050	336
rpoB	RNA polymerase, beta subunit	NIGRACIv1_280017	1211
rpoC	RNA polymerase, beta’ subunit	NIGRACIv1_280018	1317
recA	Recombinase A	NIGRACIv1_980076	272

## Author Note

Data deposition: The genome sequence reported in this paper has been deposited in the EMBL-EBI SRA archive (project number PRJEB1269), the partial nxrABC operon sequences at GenBank (accession numbers KC262217 and KC262218).
