# Supplementary material for: The Genome of Nitrospina gracilis Illuminates the Metabolism and Evolution of the Major Marine Nitrite Oxidizer
Source: Front Microbiol. 2013 Feb 21;4:27. doi: 10.3389/fmicb.2013.00027 (PMC3578206; doi:10.3389/fmicb.2013.00027)
Supplement: Supplementary Table S1 — Nitrospina gracilis strain 3/211 proteins with predicted functions in key metabolic pathways. [file 41971_Luecker_DataSheet1.DOCX]

**Table A2.** *Nitrospina gracilis* strain 3/211 proteins with predicted functions in key metabolic pathways

| Gene | Product | Identifier | Best BLAST hit in SwissProt database^a^ | Best BLAST hit in TrEMBL database^b^ | Notes |
| --- | --- | --- | --- | --- | --- |
| Nitrogen metabolism | | | | | |
| nxrA1 | Nitrite oxidoreductase, alpha subunit | NITGRv1_190001 | *Thauera selenatis* (Q9S1H0; 918/1149, 0.8, 25%, ) | *Candidatus* Kuenenia stuttgartiensis (Q1PZD8; 1148/1149, 1.0, 63%) |  |
| nxrC1 | Nitrite oxidoreductase, gamma subunit | NITGRv1_180021 | *Ideonella dechloratans* (P60000; 239/316, 0.3, 35%) | *Candidatus* Nitrospira defluvii (D8PI81; 317/316, 1.0, 41%) | 1 haem DMSO reductase-like *b*-binding domain |
| nxrA2 | Nitrite oxidoreductase, alpha subunit | NITGRv1_1060001 | *Thauera selenatis* (Q9S1H0; 918/1149, 0.8, 24%) | *Candidatus* Kuenenia stuttgartiensis (Q1PZD8; 1148/1149, 1.0, 62%) |  |
| nxrC2 | Nitrite oxidoreductase, gamma subunit | NITGRv1_1050039 | *Ideonella dechloratans* (P60000; 239/316, 0.3, 35%) | *Candidatus* Nitrospira defluvii (D8PI81; 317/316, 1.0, 41%) | 1 haem DMSO reductase-like *b*-binding domain |
| nxrB | Nitrite oxidoreductase, beta subunit | NITGRv1_1090001 | *Thauera selenatis* (Q9S1G9; 327/165, 0.3, 32%) | *Candidatus* Nitrospira defluvii (D8PI40; 429/165, 0.4, 64%) | gene fragment |
| nxrC3 | Nitrite oxidoreductase, gamma subunit | NITGRv1_550002 | *Thauera selenatis* (Q9S1G7; 239/316, 0.4, 29%) | *Candidatus* Nitrospira defluvii (D8PI81; 317/316, 1.0, 42%) | 1 haem DMSO reductase-like *b*-binding domain |
| nxrC4 | Nitrite oxidoreductase, gamma subunit | NITGRv1_570022 | *Ideonella dechloratans* (P60000; 239/266, 0.8, 28%) | *Candidatus* Nitrospira defluvii (D8PI81; 317/266, 0.9, 33%) | 1 haem DMSO reductase-like *b*-binding domain |
| alt_nxrC1 | Nitrite oxidoreductase, alternative gamma subunit | NITGRv1_920005 | *Schistocerca gregaria* (P00040; 108/576, 0.2, 30%) | *Candidatus* Nitrospira defluvii (D8PI95; 594/576, 1.0, 37%) | 2 haem *c*-binding domains |
| alt_nxrC2 | Nitrite oxidoreductase, alternative gamma subunit | NITGRv1_360061 | *Thauera selenatis* (Q9S1G7; 239/556, 0.5, 24%) | *Candidatus* Kuenenia stuttgartiensis (Q1PZE8; 535/556, 0.9, 34%) | 2 haem *c* and 1 DMSO reductase-like haem *b*-binding domains |
| alt_nxrC3 | Nitrite oxidoreductase, alternative gamma subunit | NITGRv1_420010 | *Bradyrhizobium* strain BTAi1 (A5ERU8; 206/252, 0.2, 46%) | *Candidatus* Kuenenia stuttgartiensis (Q1PVE1; 260/252, 0.4, 39%) | fragment, part 1; 1 haem *c*-binding domain |
| alt_nxrC3 | Nitrite oxidoreductase, alternative gamma subunit | NITGRv1_430001 | *Thauera selenatis* (Q9S1G7; 239/362, 0.7, 27%) | *Candidatus* Kuenenia stuttgartiensis (Q1PZE8; 535/362, 0.7, 30%) | fragment, part 2; 1 haem *c* and 1 DMSO reductase-like haem *b*-binding domain |
| alt_nxrC4 | Nitrite oxidoreductase, alternative gamma subunit | NITGRv1_620040 | *Thauera selenatis* (Q9S1G7; 239/642, 0.4, 23%) | *Candidatus* Kuenenia stuttgartiensis (Q1PZE8; 535/642, 0.7, 34%) | 3 haem *c* and 1 DMSO reductase-like haem *b*-binding domains |
|  | TorD-like Chaperone | NITGRv1_550001 | *Thauera selenatis* (Q9S1G8; 193/277, 0.3, 31%) | *Candidatus* Kuenenia stuttgartiensis (Q1PZD6; 269/277, 0.9, 34&) |  |
| nirK1 | Nitrite reductase, copper-containing | NITGRv1_100028 | *Neisseria meningitidis* (Q9JYE1; 390/336, 0.8, 55%) | *Flavobacteriaceae* bacterium (C6X0G9; 343/336, 0.9, 62%) |  |
| nirK2 | Nitrite reductase, copper-containing | NITGRv1_1050015 | *Neisseria meningitidis* (Q9JYE1; 390/355, 0.8, 47%) | *Chloroflexus aurantiacus* (A9WB08; 348/357, 0.8, 55%) |  |
| nirA | Ferredoxin-nitrite reductase | NITGRv1_160052 | *Synechococcus elongatus* (P39661; 512/542, 0.9, 44%) | *Candidatus* Nitrospira defluvii (D8PD08; 531/542, 1.0, 59%) |  |
| nirC | Nitrite transporter | NITGRv1_160053 | *Escherichia coli* (P0AC26; 268/276, 0.9, 44%) | *Candidatus* Nitrospira defluvii (D8PD05; 276/276, 1.0, 54%) |  |
| narK | Nitrite/Nitrate transporter | NITGRv1_390010 | *Arabidopsis thaliana* (Q9FJH8; 527/342, 0.3, 35%) | *Shewanella violacea* (D4ZLW9; 488/342, 0.7, 46%) | fragment |
| amtB1 | Ammonium transporter | NITGRv1_910070 | *Methanothermobacter thermautotrophicus* (O26759; 412/427, 1.0, 55%) | *Pelobacter carbinolicus* (Q3A1D0; 423/427, 1.0, 63%) |  |
| amtB2 | Ammonium transporter | NITGRv1_220003 | *Methanocaldococcus jannaschii* (Q58739; 420/410, 1.0, 40%) | *Geobacter uraniireducens* (A5G3V9; 406/410, 1.0, 67%) |  |
| Energy metabolism | | | | | |
| *Complex I* | | | | | |
| nuoM | NADH-quinone oxidoreductase, subunit M | NITGRv1_980041 | *Rickettsia conorii* (Q92G96; 493/505, 1.0, 36%) | *Candidatus* Methylomirabilis oxyfera (D5MKE2; 539/505, 0.9, 48%) |  |
| nuoL | NADH-quinone oxidoreductase, subunit L | NITGRv1_980042 | *Mycobacterium tuberculosis* (O86350; 633/623, 1.0, 42%) | *Candidatus* Kuenenia stuttgartiensis (Q1PWH4; 642/623, 1.0, 49%) |  |
| nuoK | NADH-quinone oxidoreductase, subunit K | NITGRv1_980043 | *Frankia* sp. strain CcI3 (Q2JFL0; 99/99, 0.9, 62%) | *Ktedonobacter racemifer* (D6TQP0; 106/99, 0.9, 63%) |  |
| nuoJ | NADH-quinone oxidoreductase, subunit J | NITGRv1_980044 | *Staurastrum punctulatum* (Q32S04; 181/195, 0.9, 37%) | *Rhodothermus marinus* (D0MI27; 170/195, 0.8, 38%) |  |
| nuoI | NADH-quinone oxidoreductase, subunit I | NITGRv1_980045 | *Deinococcus geothermalis* (Q1IZW5; 179/165, 0.8, 57%) | *Chloroflexus aurantiacus* (A9WFB9; 165/165, 0.8, 59%) |  |
| nuoH | NADH-quinone oxidoreductase, subunit H | NITGRv1_980046 | *Syntrophobacter fumaroxidans* (A0LJM4; 331/339, 0.9, 53%) | *Candidatus* Kuenenia stuttgartiensis (Q1PWH8; 332/339, 1.0, 57%) |  |
| nuoG | NADH-quinone oxidoreductase, subunit G | NITGRv1_980047 | *Rhizobium meliloti* (P56914; 853/826, 1.0, 31%) | *Herpetosiphon aurantiacus* (A9B502; 913/826, 1.0, 34%) |  |
| nuoF | NADH-quinone oxidoreductase, subunit F | NITGRv1_980048 | *Streptomyces coelicolor* (Q9XAQ9; 449/424, 0.9, 54%) | *Ktedonobacter racemifer* (D6TQN4; 443/424, 0.9, 56%) |  |
| nuoE | NADH-quinone oxidoreductase, subunit E | NITGRv1_980049 | *Bos taurus* (P04394; 249/158, 0.6, 47%) | *Nitrosococcus watsoni* (D8KAY9; 174/158, 0.9, 50%) |  |
| nuoD | NADH-quinone oxidoreductase, subunit D | NITGRv1_980050 | *Solibacter usitatus* (Q02CU0; 409/400, 0.9, 55%) | *Candidatus* Kuenenia stuttgartiensis (Q1PWG8; 396/400, 1.0, 57%) |  |
| nuoC | NADH-quinone oxidoreductase, subunit C | NITGRv1_980051 | *Roseiflexus* sp. strain RS-1 (A5UZH8; 174/181, 0.9, 52%) | *Parachlamydia acanthamoebae* (D1RAG7; 173/181, 0.9, 43%) |  |
| nuoB | NADH-quinone oxidoreductase, subunit B | NITGRv1_980052 | *Thermus thermophilus* (Q56218; 181/194, 0.8, 68%) | *Candidatus* Methylomirabilis oxyfera (D5MFY4; 181/194, 0.7, 74%) |  |
| nuoA | NADH-quinone oxidoreductase, subunit A | NITGRv1_980053 | *Geobacter bemidjiensis* (B5EFG3; 118/117, 1.0, 48%) | *Kribbella flavida* (D2PVR1; 119/117, 1.0, 52%) |  |
| nuoN | NADH-quinone oxidoreductase, subunit N | NITGRv1_980061 | *Geobacter lovleyi* (B3E9V6; 487/511, 1.0, 40%) | *Geobacter* sp. strain M21 (C6E8M2; 484/511, 1.0, 40%) |  |
| *Alternative complex I* | | | | | |
| nuoF | NADH-quinone oxidoreductase, subunit F | NITGRv1_410001 | *Rhizobium meliloti* (P56913; 421/608, 0.7, 39%) | *Deferribacter desulfuricans* (D3P9P1; 596/608, 1.0, 46%) | No FMN-binding site, 2 additional [4Fe-4S] and 1 [2Fe-2S] cluster |
| nuoB | NADH-quinone oxidoreductase, subunit B | NITGRv1_430003 | *Anaeromyxobacter* sp. strain Fw109-5 (A7H9V3; 183/177, 0.7, 74%) | uncultured bacterium (D6CJV8; 155/177, 0.8, 70%) |  |
| nuoN | NADH-quinone oxidoreductase, subunit N | NITGRv1_590040 | *Geobacter lovleyi* (B3E9V6; 487/475, 1.0, 48%) | *Desulfobacterales bacterium* HF0200_07G10 (E0XU59; 478/475, 1.0, 76%) |  |
| nuoM | NADH-quinone oxidoreductase, subunit M | NITGRv1_590041 | *Rhodobacter capsulatus* (P50974; 512/521, 1.0, 49%) | uncultured marine *Nitrospinaceae* bacterium (A4GJ14; 533/521, 1.0, 71%) |  |
| nuoL | NADH-quinone oxidoreductase, subunit L | NITGRv1_590042 | *Paracoccus denitrificans* (P29924; 703/656, 1.0, 42%) | uncultured marine *Nitrospinaceae* bacterium (A4GJ15; 655/656, 1.0, 73%) |  |
| nuoK | NADH-quinone oxidoreductase, subunit K | NITGRv1_590043 | *Geobacter uraniireducens* (A5G9A9; 100/100, 1.0, 61%) | *Desulfobacterales* bacterium HF0200_07G10 (E0XU56; 104/100, 1.0, 86%) |  |
| nuoJ | NADH-quinone oxidoreductase, subunit J | NITGRv1_590044 | *Paulinella chromatophora* (B1X496; 205/167, 0.8, 39%) | *Desulfobacterales* bacterium HF0200_07G10 (E0XU55; 169/167, 1.0, 76%) |  |
| nuoI | NADH-quinone oxidoreductase, subunit I | NITGRv1_590045 | *Rhodothermus marinus* (Q4QSC5; 230/189, 0.7, 39%) | *Desulfobacterales* bacterium HF0200_07G10 (E0XU54; 189/189, 1.0, 73%) |  |
| nuoH | NADH-quinone oxidoreductase, subunit H | NITGRv1_590046 | *Flavobacterium johnsoniae* (A5FKK0; 350/361, 1.0, 43%) | uncultured marine *Nitrospinaceae* bacterium (A4GJ19; 358/361, 1.0, 85%) |  |
| nuoC | NADH-quinone oxidoreductase, subunit C | NITGRv1_590047 | *Geobacter bemidjiensis* (B5EFG1; 161/160, 1.0, 50%) | uncultured marine *Nitrospinaceae* bacterium (A4GJ20; 161/160, 1.0, 68%) |  |
| nuoA | NADH-quinone oxidoreductase, subunit A | NITGRv1_590048 | *Geobacter lovleyi* (B3E9W9; 118/118, 1.0, 57%) | *Desulfobacterales* bacterium HF0200_07G10 (E0XU50; 118/118, 1.0, 75%) |  |
| nuoE | NADH-quinone oxidoreductase, subunit E | NITGRv1_810025 | *Caenorhabditis elegans* (Q20719; 239/186, 0.6, 35%) | *Anaerobaculum hydrogeniformans* (D3L0D8; 166/186, 0.8, 41%) |  |
| nuoG | NADH-quinone oxidoreductase, subunit G | NITGRv1_810026 | *Staphylococcus saprophyticus* (Q49ZN0; 984/877, 0.9, 28%) | *Candidatus* Kuenenia stuttgartiensis (Q1PWG5; 890/877, 1.0, 37%) | Additional [4Fe-4S] cluster |
| nuoD | NADH-quinone oxidoreductase, subunit D | NITGRv1_810027 | *Anaeromyxobacter* sp. strain K (B4UIA9; 431/396, 0.9, 55%) | *Anaeromyxobacter dehalogenans* (B8JH24; 431/396, 0.9, 55%) |  |
| *Complex II* | | | | | |
| sdhE | Succinate dehydrogenase, subunit C | NITGRv1_930001 | *Methanothermobacter marburgensis* (Q50755; 302/304, 0.4, 36%) | *Gloeobacter violaceus* (Q7NCN3; 298/304, 0.4, 48%) | fragment, part 1 |
| sdhE | Succinate dehydrogenase, subunit C | NITGRv1_930002 | *Methanosarcina acetivorans* (Q8TIB8; 300/186, 0.3, 35%) | *Lyngbya* sp. strain PCC 8106 (A0YKZ2; 302/196, 0.3, 54%) | fragment, part 2 |
| sdhB | Succinate dehydrogenase, Fe-S protein subunit | NITGRv1_930003 | *Proteus vulgaris* (P20921; 245/312, 0.7, 40%) | *Candidatus* Nitrospira defluvii (D8PG40; 324/312, 1.0, 44%) |  |
| sdhA | Succinate dehydrogenase, flavoprotein subunit | NITGRv1_930004 | *Rickettsia bellii* (Q1RHB9; 596/569, 1.0, 47%) | *Synechococcus* sp. strain JA-2-3B'a(2-13) (Q2JJT4; 571/569, 1.0, 53%) |  |
| sdhC | Succinate dehydrogenase, cytochrome b558 subunit | NITGRv1_1000013 | *Bacillus subtilis* (P08064; 202/227, 0.9, 33%) | *Oceanobacillus iheyensis* (Q8EPI8; 206/227, 0.9, 38%) |  |
| sdhA | Succinate dehydrogenase, flavoprotein subunit | NITGRv1_1000014 | *Bacillus subtilis* (P08065; 586/258, 0.4, 67%) | *Parachlamydia acanthamoebae* (D1R9V5; 632/258, 0.4, 68%) | fragment, part 1 |
| sdhA | Succinate dehydrogenase, flavoprotein subunit | NITGRv1_1010001 | *Bacillus subtilis* (P08065; 586/346, 0.6, 46%) | *Stigmatella aurantiaca* (Q08WU1; 625/346, 0.6, 46%) | fragment, part 2 |
| sdhB | Succinate dehydrogenase, iron-sulfur protein subunit | NITGRv1_1010002 | *Bacillus subtilis* (P08066; 253/255, 1.0, 63%) | *Staphylococcus aureus* (C8MEK7; 271/255, 0.9, 63%) |  |
| *Complex III* | | | | | |
| qcrB | Ubiquinol-cytochrome c reductase, cytochrome b subunit | NITGRv1_570009 | *Heliobacillus mobilis* (Q9ZGG0; 213/353, 0.5, 38%) | *Nocardioides* sp. strain BAA-499 (A1SKW1; 342/353, 1.0, 45%) |  |
| petC | Ubiquinol-cytochrome c reductase, Rieske 2Fe-2S iron sulfur subunit | NITGRv1_700035 | *Chlorobium tepidum* (Q9F722; 181/187, 0.7, 36%) | *Candidatus* Methylomirabilis oxyfera (D5MG49; 197/187, 0.8, 59%) |  |
| petB | Ubiquinol-cytochrome c reductase, cytochrome b subunit | NITGRv1_700036 | *Coleochaete orbicularis* (Q71KQ6; 215/274, 0.7, 45%) | *Candidatus* Methylomirabilis oxyfera (D5MG48; 256/274, 0.9, 80%) |  |
| petD | Ubiquinol-cytochrome c reductase, cytochrome b subunit | NITGRv1_700037 | *Lepilemur septentrionalis* (Q20FQ1; 379/270, 0.3, 31%) | *Candidatus* Methylomirabilis oxyfera (D5MG47; 269/270, 1.0, 59%) |  |
|  | Octaheam cytochrome c | NITGRv1_700039 | *Pseudomonas stutzeri* (Q52527; 146/767, 0.1, 30%) | *Candidatus* Methylomirabilis oxyfera (D5MG46; 856/767, 0.9, 35%) | Conserved in *Ca.* K. stuttgartiensis and *Ca.* M. oxyfera complex III |
| *Complex IV* | | | | | |
| ccoNOP | Cytochrome c oxidase | NITGRv1_270015 | *Rhizobium meliloti* (Q05572; 539/954, 0.5, 30%) | *Psychroflexus torquis* (Q1VRL1; 734/954, 0.7, 33%) | *cbb_3_*-type, fused subunits I, II, and III |
| bd*-like terminal oxidase* | | | | | |
|  | Cytochrome *bd*-type quinol oxidase subunit 1-like protein | NITGRv1_920008 | *Azotobacter vinelandii* (Q09049; 537/661, 0.2, 26%) | *Candidatus* Nitrospira defluvii (D8PI98; 639/661, 0.9, 60%) | part of NXR module, unknown function |
|  | Cytochrome bd-type quinol oxidase subunit 1-like protein | NITGRv1_750009 | *Xenopus laevis* (P35616; 544/746, 0.1, 44%) | *Candidatus* Nitrospira defluvii (D8PBQ1; 567/746, 0.7, 38%) |  |
|  |  |  |  |  |  |
| *Complex V* | | | | | |
| atpC | ATP synthase, F1 complex, epsilon subunit | NITGRv1_870005 | *Pelobacter carbinolicus* (Q39ZU2; 138/141, 0.9, 43%) | *Desulfuromonas acetoxidans* (Q1JZG9; 137/141, 0.9, 42%) |  |
| atpD | ATP synthase, F1 complex, beta subunit | NITGRv1_870006 | *Carboxydothermus hydrogenoformans* (Q3A946; 473/470, 1.0, 74%) | *Deferribacter desulfuricans* (D3P9E8; 468/470, 1.0, 77%) |  |
| atpG | ATP synthase, F1 complex, gamma subunit | NITGRv1_870007 | *Chlorobaculum parvum* (B3QL62; 292/285, 1.0, 49%) | *Deferribacter desulfuricans* (D3P9E9; 285/285, 1.0, 50%) |  |
| atpA | ATP synthase, F1 complex, alpha subunit | NITGRv1_870008 | *Heliobacterium modesticaldum* (B0THN4; 502/501, 1.0, 74%) | *Deferribacter desulfuricans* (D3P9F0; 502/501, 1.0, 74%) |  |
| atpH | ATP synthase, F1 complex, delta subunit | NITGRv1_870009 | *Myxococcus xanthus* (Q1CWT4; 182/184, 1.0, 27%) | *Thermoanaerobacter italicus* (D3T7J4; 177/184, 0.9, 31%) |  |
| atpF | ATP synthase, F0 complex, subunit B | NITGRv1_870010 | *Leptospira interrogans* (Q72SY3; 173/170, 0.9, 33%) | *Candidatus* Nitrospira defluvii (D8P7R4; 170/170, 1.0, 39%) |  |
| atpE | ATP synthase, F0 complex, subunit C | NITGRv1_870011 | *Magnetospirillum magneticum* (Q2W027; 74/75, 1.0, 53%) | *Candidatus* Nitrospira defluvii (D8P7R5; 76/75, 1.0, 71%) |  |
| atpB | ATP synthase, F0 complex, subunit A | NITGRv1_870012 | *Magnetococcus* sp. strain MC-1 (A0LDW6; 259/199, 0.7, 54%) | *Candidatus* Nitrospira defluvii (D8P7R6; 249/199, 0.8, 59%) | fragment, part 2 |
| atpB | ATP synthase, F0 complex, subunit A | NITGRv1_870013 | *Magnetococcus* sp. strain MC-1 (A0LDW6; 259/45, 0.2, 44%) | *Candidatus* Nitrospira defluvii (D8P7R6; 249/45, 0.2, 63%) | fragment, part 1 |
| atpI | ATP synthase, subunit I | NITGRv1_870014 | *Rhodospirillum rubrum* (P15011; 123/98, 0.6, 37%) | *Magnetospirillum magneticum* (Q2W029; 122/98, 0.5, 45%) |  |
| ppa | Inorganic pyrophosphatase | NITGRv1_130065 | *Chlamydomonas reinhardtii* (Q949J1; 192/180, 0.9, 63%) | *Volvox carteri* (D8U7Y6; 192/180, 0.9, 63%) |  |
| *Etf-quinone reductase* | | | | | |
| etfA | Electron transfer flavoprotein, alpha subunit | NITGRv1_190030 | *Bacillus subtilis* (P94551; 325/331, 1.0, 44%) | *Desulfitobacterium hafniense* (Q24S32; 328/331, 1.0, 49%) |  |
| etfB | Electron transfer flavoprotein, beta subunit | NITGRv1_190036 | *Bacillus subtilis* (P94550; 257/262, 1.0, 38%) | *Desulfitobacterium hafniense* (B8FWN8; 260/262, 1.0, 48%) |  |
| hbd | 3-hydroxybutyryl-CoA dehydrogenase | NITGRv1_290054 | *Thermoanaerobacterium thermosaccharo-lyticum* (P77851; 281/300, 0.9, 47%) | *Anaeromyxobacter* sp. strain Fw109-5 (A7HC91; 284/300, 0.9, 52%) |  |
| bcd | Acyl-CoA dehydrogenase, short-chain specific | NITGRv1_290055 | *Bacillus subtilis* (O34421; 380/442, 0.8, 33%) | *Anaerococcus tetradius* (C2CKL9; 378/452, 0.8, 32%) |  |
| *Sulfhydrogenase* | | | | | |
| hypE | Hydrogenase maturation protein HypE | NITGRv1_190060 | *Azotobacter chroococcum* (P42034; 341/369, 0.9, 52%) | *Cyanothece* sp. strain PCC 8801 (B7JZ80; 348/369, 0.9, 61%) | Carbamoyl phosphate phosphatase |
| hypD | Hydrogenase maturation protein HypD | NITGRv1_190061 | *Rhodobacter capsulatus* (P26411; 377/361, 1.0, 49%) | *Rhodothermus marinus* (D0MFX9; 369/361, 1.0, 70%) |  |
| hypC | Hydrogenase assembly chaperone HypC | NITGRv1_190062 | *Synechocystis* sp. strain PCC 6803 (P74095; 76/86, 0.9, 55%) | *Rhodothermus marinus* (D0MFX8; 85/86, 1.0, 59%) |  |
| hypF | Hydrogenase maturation protein HypF | NITGRv1_190063 | *Synechocystis* sp. strain PCC 6803 (Q55638; 767/821, 1.0, 41%) | *Pedosphaera parvula* (B9XAS8; 791/821, 1.0, 50%) | Carbamoyltransferase |
| hypA | Hydrogenase nickel incorporation protein HypA | NITGRv1_190064 | *Desulfitobacterium hafniense* (B8G0K1; 114/98, 0.6, 35%) | *Verrucomicrobiae* bacterium DG1235 (B5JEM0; 85/98, 0.9, 50%) |  |
| hycI | Hydrogenase maturation protease HycI | NITGRv1_190065 | *Azotobacter chroococcum* (Q43954; 209/162, 0.6, 32%) | *Magnetospirillum gryphiswaldense* (A4U2E7; 153/162, 0.9, 43%) |  |
| hydA | Nickel-dependent hydrogenase, alpha subunit | NITGRv1_190066 | *Methanococcus voltae* (Q00404; 474/430, 1.0, 29%) | *Rhodothermus marinus* (D0MFX4; 433/430, 1.0, 63%) | group 3b [NiFe]-hydrogenase |
| hydD | Nickel-dependent hydrogenase, delta subunit | NITGRv1_190067 | *Methanocaldococcus jannaschii* (Q58591; 288/275, 0.8, 34%) | *Methylosinus trichosporium* (D5QQB6; 260/275, 0.9, 65%) |  |
| hydC | Nickel-dependent hydrogenase, gamma subunit | NITGRv1_190068 | *Salmonella typhimurium* (P26475; 272/282, 0.9, 31%) | *Rhodothermus marinus* (D0MFX2; 287/282, 0.9, 62%) |  |
|  | Hydrogenase accessory protein | NITGRv1_190069 | *Bos taurus* (P00516; 671/160, 0.1, 30%) | *Azotobacter vinelandii* (C1DJA8; 152/160, 0.9, 53%) | Cyclic nucleotide-binding protein |
| hydB | Nickel-dependent hydrogenase, beta subunit | NITGRv1_190070 | *Salmonella typhimurium* (P26474; 347/387, 0.7, 29%) | *Cyanothece* sp. strain PCC 7425 (B8HJR4; 391/387, 1.0, 63%) |  |
| Carbon metabolism | | | | | |
| *Citric acid cycle/carbon fixation* | | | | | |
| cynT | Carbonic anhydrase | NITGRv1_470004 | *Mycobacterium tuberculosis* (O53573; 207/190, 0.9, 39%) | uncultured *Desulfobacterium* sp. (E1Y880; 220/190, 0.8, 47%) |  |
| acsA | Acetyl-coenzyme A synthetase | NITGRv1_750002 | *Magnetococcus* sp. strain MC-1 (A0L576; 644/657, 1.0, 67%) | *Desulfarculus baarsii* (E1QLV5, 665/657, 1.0, 67%) |  |
| porE | Pyruvate:ferredoxin oxidoreductase, epsilon subunit | NITGRv1_90066 | *Aquifex aeolicus* (O67232; 78/93, 0.6, 40%) | *Candidatus* Poribacteria sp. WGA-A3 (D2MI73; 92/93, 1.0, 62%) | 2 [4Fe-4S] clusters |
| porC | Pyruvate:ferredoxin oxidoreductase, gamma subunit | NITGRv1_90067 | *Pyrococcus horikoshii* (O58411; 185/247, 0.7, 38%) | *Leptospirillum rubarum* (A3EQL8; 232/247, 0.9, 71%) | catalytic domain |
| porB | Pyruvate:ferredoxin oxidoreductase, beta subunit | NITGRv1_90068 | *Methanosarcina barkeri* (P80522; 296/300, 0.9, 32%) | *Candidatus* Poribacteria sp. WGA-A3 (D2MI71; 306/300, 1.0, 77%) | Thiamine pyrophosphate (TPP) binding |
| porA | Pyruvate:ferredoxin oxidoreductase, alpha subunit | NITGRv1_90069 | *Methanothermobacter marburgensis* (P80900; 383/417, 0.9, 33%) | *Candidatus* Nitrospira defluvii (D8PBX4; 403/417, 1.0, 75%) | Transketolase |
| porD | Pyruvate:ferredoxin oxidoreductase, delta subunit | NITGRv1_90070 | *Nematostella vectensis* (A7SNN5; 978/213, 0.1, 27%) | *Leptospirillum rubarum* (A3EQD4; 193/213, 0.9, 70%) |  |
| pdhA | Pyruvate dehydrogenase, E1 component, alpha subunit | NITGRv1_160039 | *Bacillus subtilis* (O31404; 333/313, 0.9, 39%) | *Beggiatoa* sp. PS (A7BPK6; 331/313, 0.9, 48%) |  |
| pdhB | Pyruvate dehydrogenase, E1 component, beta subunit | NITGRv1_160040 | *Cupriavidus necator* (P27746; 338/353, 0.9, 43%) | delta proteobacterium NaphS2 (D8F7A2; 364/353, 0.9, 55%) |  |
| pdhA | Pyruvate dehydrogenase, E1 component, subunit alpha | NITGRv1_170041 | *Porphyra yezoensis* (Q1XDM0; 346/341; 0.9, 37%) | *Clostridium cellulolyticum* (B8I1V3; 321/341, 0.9, 47%) |  |
| pdhB | Pyruvate dehydrogenase, E1 component, subunit beta | NITGRv1_170042 | *Bacillus subtilis* (P37941; 327/346, 0.9, 40%) | delta proteobacterium NaphS2 (D8F7A2; 364/346, 0.9, 57%) |  |
| aceE | Pyruvate dehydrogenase, E1 component | NITGRv1_1060005 | *Cupriavidus necator* (Q59097; 895/326, 0.3, 65%) | *Nitrosococcus halophilus* (D5BYH5; 891/326, 0.3, 72%) | fragment, part 1 |
| aceE | Pyruvate dehydrogenase, E1 component | NITGRv1_1060006 | *Pseudomonas aeruginosa* (Q59637; 882/310, 0.3, 56%) | *Nitrosococcus halophilus* (D5BYH5; 891/310, 0.3, 70%) | fragment, part 2 |
| aceE | Pyruvate dehydrogenase, E1 component | NITGRv1_1060007 | *Pseudomonas aeruginosa* (Q59637; 882/288, 0.3, 58%) | *Acidobacterium capsulatum* (C1F9X9; 880/288, 0.3, 61%) | fragment, part 3 |
| aceF | putative Pyruvate/2-oxoglutarate dehydrogenase complex, dihydrolipoamide acyltransferase (E2) component | NITGRv1_1060008 | *Pseudomonas aeruginosa* (Q59638; 547/289, 0.5, 30%) | *Gramella forsetii* (A0M206; 507/289, 0.7, 33%) | fragment part 1 |
| aceF | putative Pyruvate/2-oxoglutarate dehydrogenase complex, dihydrolipoamide acyltransferase (E2) component | NITGRv1_1060009 | *Azotobacter vinelandii* (P10802; 638/172, 0.3, 51%) | *Nitrosococcus halophilus* (D5BYH6; 441/172, 0.4, 56%) | fragment part 2 |
| lpdA | Pyruvate/2-oxoglutarate dehydrogenase complex, dihydrolipoyl dehydrogenase (E3) component | NITGRv1_1060010 | *Escherichia coli* (P0A9P0; 474/463, 1.0, 47%) | *Planctomyces maris* (A6C4P3; 475/463, 1.0, 61%) |  |
|  | Dihydrolipoyl dehydrogenase | NITGRv1_20031 | *Staphylococcus aureus* (P0A0E5; 547/457, 0.8, 33%) | *Candidatus* Nitrospira defluvii (D8P919; 453/457, 1.0, 55%) |  |
|  | Dihydrolipoyl dehydrogenase | NITGRv1_700034 | *Halobacterium salinarum* (Q9HN74; 474/448, 1.0, 28%) | *Thermosediminibacter oceani* (D9S2E4; 474/448, 0.9, 31%) |  |
| lpd | Dihydrolipoyl dehydrogenase | NITGRv1_750015 | *Vibrio cholerae* (Q9KPF6; 475/464, 1.0, 47%) | *Planctomyces maris* (A6C4P3; 475/464, 1.0, 56%) |  |
| oadA | Oxaloacetate decarboxylase, alpha subunit | NITGRv1_90090 | *Methanocaldococcus jannaschii* (Q58628; 567/502, 0.8, 61%) | *Vibrio shilonii* (A6D844; 595/502; 0.9, 58%) | fragment, part 1 |
| oadA | Oxaloacetate decarboxylase, alpha subunit | NITGRv1_100001 | *Bacillus subtilis* (Q9KWU4; 1148/41, 0.0, 53%) | *Spirochaeta smaragdinae* (E1R9D2; 600/41, 0.1, 56%) | fragment, part 2 |
| oadB | Oxaloacetate decarboxylase, beta subunit | NITGRv1_100002 | *Acidaminococcus fermentans* (Q9ZAA6; 375/404, 0.9, 64%) | *Caldicellulosiruptor saccharolyticus* (A4XMC4; 378/404, 0.9, 67%) |  |
| oadG | Oxaloacetate decarboxylase, gamma subunit | NITGRv1_100003 | *Vibrio cholerae* (Q9KTU4; 90/92, 0.9, 35%) | *Allochromatium vinosum* (D3RVM7; 82/92, 0.8, 36%) |  |
| ppdK | Pyruvate, phosphate dikinase | NITGRv1_250092 | *Rhizobium meliloti* (Q59754; 898/879, 1.0, 56%) | unc. marine crenarchaeote HF4000_ APKG8D22 (B3TAD5; 876/879, 1.0, 71%) |  |
| pckA | Phosphoenolpyruvate carboxykinase (ATP) | NITGRv1_100046 | *Geobacter uraniireducens* (A5GBF9; 530/547, 0.9, 63%) | *Allochromatium vinosum* (D3RSB3; 533/547, 1.0, 61%) |  |
| mdh | Malate dehydrogenase | NITGRv1_170079 | *Chloroflexus aggregans* (B8GDA2; 309/328, 0.7, 58%) | *Chlorobium ferrooxidans* (Q0YS54; 310/328, 0.7, 63%) |  |
| fumC | Fumarate hydratase | NITGRv1_170099 | *Leptospira interrogans* (Q8F9L0; 464/462, 1.0, 69%) | *Rhodospirillum centenum* (B6INS7; 464/462, 1.0, 69%) | class II |
| sucC | Succinyl-CoA synthetase, beta subunit | NITGRv1_910003 | *Desulfatibacillum alkenivorans* (B8FLW6; 386/386, 1.0, 63%) | *Bacillus* sp. NRRL B-14911 (Q2B7D9; 386/386, 1.0, 62%) |  |
| sucD | Succinyl-CoA synthetase, alpha subunit | NITGRv1_910004 | *Staphylococcus aureus* (Q5HGI6; 302/289, 0.9, 64%) | *Chlorobaculum parvum* (B3QQF4; 300/289, 1.0, 67%) |  |
| forA | 2-oxoglutarate:ferredoxin oxidoredutase, alpha subunit | NITGRv1_910009 | *Methanothermobacter thermautotrophicus* (P56810; 383/404, 0.8, 32%) | *Leptospirillum ferrodiazotrophum* (C6HVM2; 414/404, 0.9, 69%) |  |
| forB | 2-oxoglutarate:ferredoxin oxidoredutase, beta subunit | NITGRv1_910010 | *Methanosarcina barkeri* (P80522; 296/289, 0.7, 29%) | *Leptospirillum rubarum* (A3EQL1; 288/289, 1.0, 73%) |  |
| forC | 2-oxoglutarate:ferredoxin oxidoredutase, gamma subunit | NITGRv1_910011 | *Pyrococcus furiosus* (Q51799; 185/237, 0.7, 33%) | *Leptospirillum* sp. Group II '5-way CG' (B6ANA7; 232/237, 0.9, 70%) |  |
| forE | 2-oxoglutarate:ferredoxin oxidoredutase, epsilon subunit | NITGRv1_910012 | *Aquifex aeolicus* (O67232; 78/87, 0.8, 49%) | *Leptospirillum* sp. Group II '5-way CG' (B6ANA8; 101/87, 0.8, 55%) |  |
| idh | Isocitrate dehydrogenase [NADP] | NITGRv1_1060015 | *Azotobacter vinelandii* (P16100; 741/743, 1.0, 63%) | *Magnetococcus* sp. strain MC-1 (A0L819; 742/743, 1.0, 67%) |  |
| acnA | Aconitate hydratase | NITGRv1_710028 | *Dictyostelium discoideum* (Q54XS2; 771/755, 1.0, 55%) | *Leptospira interrogans* (Q8EX87; 757/755, 1.0, 59%) |  |
| prpC | 2-methylcitrate synthase/citrate synthase type I | NITGRv1_290045 | *Bacillus subtilis* (P39120; 372/398, 1.0, 38%) | *Geobacillus* sp. strain Y412MC61 (C9RTJ2; 372/398, 1.0, 39%) |  |
| gltA | Citrate (Si)-synthase | NITGRv1_810009 | *Mycobacterium tuberculosis* (Q10530; 431/431, 1.0, 57%) | *Rhodothermus marinus* (D0MDV9; 425/431, 1.0, 61%) | Citrate synthase, type II |
|  | ATP citrate lyase, beta subunit | NITGRv1_580013 | *Desulfatibacillum alkenivorans* (B8FLW6; 386/422, 0.7, 26%) | *Magnetococcus* sp. strain MC-1 (A0LDT1; 424/422, 1.0, 73%) |  |
|  | ATP citrate lyase, alpha subunit | NITGRv1_580014 | *Aquifex aeolicus* (O67547; 305/904, 0.4, 32%) | *Magnetococcus* sp. strain MC-1 (A0LDT0; 916/904, 1.0, 62%) |  |
| *Glycolysis/Gluconeogenesis* | | | | | |
| algC | Phosphomannomutase/phosphoglucomutase | NITGRv1_100071 | *Pseudomonas aeruginosa* (P26276; 463/457, 1.0, 45%) | uncultured *Desulfobacterium* sp. (E1YC29; 450/457, 1.0, 53%) |  |
| glk | Glucokinase | NITGRv1_830004 | *Synechocystis* sp. strain PCC 6803 (Q55855; 355/311, 0.1, 44%) | *Gallionella capsiferriformans* (D9SHB8; 329/51, 0.1, 50%) | fragment, part 2 |
| glk | Glucokinase | NITGRv1_840001 | *Nostoc punctiforme* (B2J224; 341/253, 0.8, 35%) | *Pedosphaera parvula* (B9XN74; 336/253, 0.8, 41%) | fragment, part 1 |
|  | Transaldolase | NITGRv1_840002 | *Prochlorococcus marinus* (Q31C15; 333/416, 0.6, 30%) | *Methylacidiphilum infernorum* (B3DVG6; 491/416, 0.9, 28%) |  |
| pgi | Glucose-6-phosphate isomerase | NITGRv1_840003 | *Parabacteroides distasonis* (A6L8C4; 447/436, 1.0, 57%) | *Parabacteroides merdae* (A7AHR8; 447/436, 1.0, 57%) |  |
| scrK | Fructokinase | NITGRv1_950073 | *Pyrococcus horikoshii* (O59128; 310/305, 0.9, 26%) | *Desulfococcus oleovorans* (A8ZUP6; 308/305, 1.0, 39%) |  |
| fbp | Fructose-1,6-bisphosphatase class 1 | NITGRv1_740031 | *Desulfovibrio salexigens* (C6BTU7; 338/329, 1.0, 56%) | delta proteobacterium NaphS2 (D8F263; 344/329, 1.0, 55%) |  |
| pfkA | 6-phosphofructokinase | NITGRv1_250013 | *Chloroflexus aggregans* (B8GAA4; 356/364, 1.0, 50%) | *Desulfomicrobium baculatum* (C7LNY9; 367/364, 1.0, 65%) |  |
| fba | Fructose-biphosphate aldolase | NITGRv1_680018 | *Escherichia coli* (B7MEF1; 284/466, 0.6, 26%) | *Candidatus* Kuenenia stuttgartiensis (Q1PYR9; 462/466, 1.0, 56%) |  |
| tpiA | Triosephosphate isomerase | NITGRv1_700030 | *Geobacter metallireducens* (Q39U97; 251/251, 1.0, 50%) | *Planctomyces maris* (A6CEM4; 257/251, 1.0, 51%) |  |
| serA | D-3-phosphoglycerate dehydrogenase | NITGRv1_360082 | *Archaeoglobus fulgidus* (O29445; 527/526, 1.0, 47%) | *Syntrophobacter fumaroxidans* (A0LPG7; 526/526, 1.0, 50%) |  |
| gapA | Glyceraldehyde-3-phosphate dehydrogenase | NITGRv1_700029 | *Bacillus cereus* (Q4MQ58; 334/339, 1.0, 65%) | *Carboxydothermus hydrogenoformans* (Q3AFD2; 335/339, 1.0, 66%) |  |
|  | Phosphoglycerate kinase | NITGRv1_710009 | *Dictyoglomus turgidum* (B8E2D3; 396/520, 1.0, 31%) | *Anaerococcus prevotii* (C7RH33; 398/520, 0.9, 31%) |  |
| apgM | Phosphoglycerate mutase, 2,3-bisphosphoglycerate-independent | NITGRv1_130028 | *Geobacter sulfurreducens* (Q74C57; 399/404, 1.0, 37%) | *Geobacter sulfurreducens* (D7AJP9; 399/404, 1.0, 37%) |  |
| gpmA | Phosphoglycerate mutase, 2,3-bisphosphoglycerate-dependent | NITGRv1_200010 | *Prosthecochloris aestuarii* (B4S616; 247/248, 1.0, 70%) | *Chlorobium ferrooxidans* (Q0YS24; 247/248, 1.0, 71%) |  |
|  | Phosphoglycerate mutase | NITGRv1_490020 | *Enterobacter* sp. strain 638 (A4W6B3; 215/241, 0.8, 32%) | *Geobacter bemidjiensis* (B5EEL7; 198/241, 0.8, 42%) |  |
| eno | Enolase | NITGRv1_750034 | *Geobacter* sp. strain FRC-32 (B9M3M1; 430/161, 0.4, 74%) | *Geobacter sulfurreducens* (D7AMA9; 428/161, 0.4, 75%) | fragment, part 2 |
| eno | Enolase | NITGRv1_750035 | *Geobacillus thermodenitrificans* (A4ISP4; 430/276, 0.6, 70%) | *Geobacillus* sp. G11MC16 (B4BN43; 430/276, 0.6, 70%) | fragment, part 1 |
|  | Phosphoenolpyruvate mutase | NITGRv1_950026 | *Tetrahymena pyriformis* (P33182; 300/589, 0.5, 37%) | *Sorangium cellulosum* (A9EXQ4; 554/589, 1.0, 34%) |  |
| *Pentose phosphate pathway* | | | | | |
| pgl | 6-phosphogluconolactonase | NITGRv1_20001 | *Aggregatibacter actinomycetemcomitans* (P70715; 232/355, 0.5, 43%) | *Sphaerobacter thermophilus* (D1C7Y2; 252/355, 0.4, 53%) |  |
| zwf | Glucose-6-phosphate 1-dehydrogenase | NITGRv1_20002 | *Aggregatibacter actinomycetemcomitans* (P77809; 494/510, 1.0, 54%) | sediment metagenome (D9PMN5; 507/510, 1.0, 56%) |  |
| gnd | 6-phosphogluconate dehydrogenase (decarboxylating) | NITGRv1_20003 | *Bacillus subtilis* (P54448; 297/298, 1.0, 43%) | *Sphaerobacter thermophilus* (D1C7X9; 301/298, 1.0, 56%) |  |
| rpiB | Ribose-5-phosphate isomerase B | NITGRv1_720014 | *Escherichia coli* (P37351; 149/159, 0.9, 46%) | *Thermodesulfovibrio yellowstonii* (B5YFY9; 150/159, 0.9, 58%) |  |
| rpe | D-ribulose-5-phosphate 3-epimerase | NITGRv1_590035 | *Bacillus subtilis* (O34557; 217/215, 1.0, 62%) | unc. *Desulfobacterales* bacterium HF0200_ 07G10 (E0XU63; 255/215, 0.8, 67%) |  |
| prsA | Ribose-phosphate pyrophosphokinase | NITGRv1_250058 | *Desulfotalea psychrophila* (Q6AJL7; 313/320, 1.0, 61%) | *Geobacter bemidjiensis* (B5EHX3; 316/320, 1.0, 65%) |  |
| tktA | Transketolase | NITGRv1_730002 | *Methanocaldococcus jannaschii* (Q58092; 316/313, 0.9, 45%) | *Thermoanaerobacter italicus* (D3T4D7; 306/313, 1.0, 55%) | fragment, part 2 |
| tktA | Transketolase | NITGRv1_730003 | *Methanocaldococcus jannaschii* (Q58094; 274/278, 1.0, 52%) | *Anaerocellum thermophilum* (B9MLE7; 282/278, 0.9, 56%) | fragment, part 1 |
|  | Transaldolase | NITGRv1_840002 | *Prochlorococcus marinus* (Q31C15; 333/416, 0.6, 30%) | *Methylacidiphilum infernorum* (B3DVG6; 491/416, 0.9, 28%) |  |
| *Glycogen metabolism* | | | | | |
| glgC | Glucose-1-phosphate adenylyltransferase | NITGRv1_1040023 | *Thermus thermophilus* (Q5SMC1; 414/411, 1.0, 53%) | *Candidatus* Methylomirabilis oxyfera (D5MLA3; 417/411, 1.0, 56%) |  |
| glgA | Glycogen synthase | NITGRv1_590026 | *Anaeromyxobacter dehalogenans* (Q2IM81; 475/489, 1.0, 47%) | *Candidatus* Methylomirabilis oxyfera (D5MJP6; 488/489, 1.0, 49%) |  |
| glgP | Alpha-glucan phosphorylase | NITGRv1_360059 | *Mycobacterium tuberculosis* (Q10639; 863/582, 0.7, 39%) | *Pelobacter carbinolicus* (Q3A3T3; 570/582, 1.0, 53%) |  |
| glgP | Glycogen phosphorylase | NITGRv1_660004 | *Homo sapiens* (P11217; 842/828, 1.0, 55%) | *Candidatus* Kuenenia stuttgartiensis (Q1Q798; 831/828, 1.0, 60%) |  |
|  | 4-alpha-glucanotransferase | NITGRv1_250098 | *Dictyoglomus thermophilum* (P09961; 686/722, 1.0, 40%) | *Dethiobacter alkaliphilus* (C0GEN2; 691/722, 1.0, 43%) | glycogen debranching enzyme |
|  | UTP--glucose-1-phosphate uridylyltransferase | NITGRv1_660005 | *Pyrus pyrifolia* (O64459; 471/455, 0.9, 43%) | *Selaginella moellendorffii* (D8RTW0; 475/455, 0.9, 43%) |  |
| susA | Sucrose synthase | NITGRv1_950071 | *Vicia faba* (P31926; 806/811, 0.9, 47%) | *Desulfurivibrio alkaliphilus* (D6Z3A6; 797/811, 1.0, 49%) |  |
| sps | Sucrose-phosphate synthase | NITGRv1_950072 | *Zea mays* (P31927; 1068/720, 0.5, 42%) | *Desulfococcus oleovorans* (A8ZUP7; 735/720, 1.0, 58%) |  |
| ugd | UDP-glucose 6-dehydrogenase | NITGRv1_750029 | *Glycine max* (Q96558; 480/460, 1.0, 58%) | *Victivallis vadensis* (D1N2T5; 457/460, 1.0, 65%) |  |
| Sulfur metabolism | | | | | |
| sorB | Sulfite:cytochrome c oxidoreductase, subunit B | NITGRv1_140025 | *Rattus norvegicus* (Q9ESS6; 624/127, 0.1, 38%) | uncultured bacterium Ak20-3 (D9MX74; 126/127, 0.6, 53%) |  |
| sorA | Sulfite:cytochrome c oxidoreductase, subunit A | NITGRv1_140026 | *Rattus norvegicus* (Q07116; 546/435, 0.8, 34%) | *Nitrosococcus halophilus* (D5BZZ4; 440/435, 1.0, 59%) |  |
| cysD | Sulfate adenylyltransferase, subunit 2 | NITGRv1_100065 | *Vibrio fischeri* (Q5E831; 302/267, 1.0, 43%) | *Geobacter uraniireducens* (A5G862; 265/267, 1.0, 70%) |  |
| cysNC | Sulfate adenylyltransferase, subunit 1, and adenylylsulfate kinase | NITGRv1_100066 | *Rhizobium* sp. strain N33 (P72339; 646/608, 0.8, 37%) | *Geobacter* sp. strain FRC-32 (B9M546; 619/608, 1.0, 53%) | bifunctional enzyme |
|  | Adenylyl-sulfate reductase (thioredoxin) | NITGRv1_620035 | *Arabidopsis thaliana* (P92981; 454/229, 0.5, 51%) | *Candidatus* Methylomirabilis oxyfera (D5MMZ5; 261/229, 0.8, 62%) |  |
| sir | Ferredoxin-sulfite reductase | NITGRv1_810017 | *Oryza sativa* (Q42997; 596/799, 0.6, 28%) | *Candidatus* Nitrospira defluvii (D8PAC5; 847/799, 1.0, 42%) |  |
|  | Sulfate permease | NITGRv1_250078 | *Bacillus subtilis* (O06984; 530/580, 0.9, 30%) | *Nitratiruptor* sp. strain SB155-2 (A6Q1R5; 545/580, 0.9, 51%) |  |
|  | Sulfate permease | NITGRv1_470006 | *Bacillus subtilis* (O06984; 530/599, 0.9, 31%) | *Nitratiruptor* sp. strain SB155-2 (A6Q1R5; 545/599, 0.9, 58%) |  |
|  | Sulfate transporter | NITGRv1_1060037 | *Bacillus subtilis* (O06984; 530/564, 1.0, 30%) | *Nitratiruptor* sp. strain SB155-2 (A6Q1R5; 545/564, 0.9, 52%) |  |
| Siderophore biosynthesis | | | | | |
| sbnA | Siderophore biosynthesis protein SbnA | NITGRv1_360017 | *Ralstonia solanacearum* (Q8XSQ0; 338/341, 1.0, 58%) | *Nitrobacter* sp. Nb-311A (A3WU42; 339/341, 0.9, 61%) |  |
| sbnB | Siderophore biosynthesis protein SbnB | NITGRv1_360018 | *Rhizobium meliloti* (P58339; 330/347, 0.8, 26%) | *Ralstonia solanacearum* (D8P2U4; 339/347, 1.0, 66%) | Ornithine cyclodeaminase |
| sbnC | Siderophore biosynthesis protein SbnC | NITGRv1_360019 | *Rhizobium* sp. strain NGR234 (P55706; 628/640, 0.9, 22%) | *Dickeya dadantii* (D2C344; 630/640, 0.8, 36%) |  |
| sbnD | Siderophore biosynthesis protein SbnD | NITGRv1_360020 | *Bacillus subtilis* (P40760; 392/401, 0.9, 29%) | *Acinetobacter* sp. SH024 (D6JTG1; 401/401, 1.0, 39%) | MFS-type |
| sbnE | Siderophore biosynthesis protein SbnE | NITGRv1_360021 | *Rhizobium meliloti* (Q9Z3R0; 585/599, 1.0, 26%) | *Nitrobacter* sp. Nb-311A (A3WU36; 574/599, 1.0, 37%) |  |
| sbnF | Siderophore biosynthesis protein SbnF | NITGRv1_360022 | *Rhizobium meliloti* (Q9Z3Q7; 601/628, 0.9, 32%) | *Nitrobacter* sp. Nb-311A (A3WU37 (593/628, 0.9, 46%) |  |
| sbnG | Siderophore biosynthesis protein SbnG | NITGRv1_360023 | *Enterobacter* sp. strain 638 (A4WEU6; 256/265, 0.9, 30%) | *Dickeya dadantii* (E0SIF4; 258/265, 1.0, 56%) | alpha-dehydro-beta-deoxy-D-glucarate aldolase |
| sbnH | Siderophore biosynthesis protein SbnH | NITGRv1_360024 | *Vibrio cholerae* (Q9KVL7; 417/417, 0.9, 31%) | *Geobacillus* sp. strain C56-T3 (D7D019; 401/417, 0.9, 52%) | Diaminopimelate decarboxylase |
| Resistance and defense | | | | | |
| *Sodium extrusion* | | | | | |
| mnhE | Monovalent cation/H+ antiporter, subunit E | NITGRv1_160007 | *Rhizobium meliloti* (Q52982; 161/168, 0.9, 31%) | *Candidatus* Poribacteria sp. WGA-A3 (D2MI02; 177/168, 1.0, 59%) |  |
| mnhF | Monovalent cation/H+ antiporter, subunit F | NITGRv1_160008 | *Bacillus subtilis* (O05228; 94/88, 0.9, 33%) | *Candidatus* Poribacteria sp. WGA-A3 (D2MI03; 79/88, 0.9, 87%) |  |
| mnhG | Monovalent cation/H+ antiporter, subunit G | NITGRv1_160009 | *Bacillus subtilis* (O05227; 124/99, 0.7, 40%) | *Candidatus* Poribacteria sp. WGA-A3 (D2MI04; 99/99, 1.0, 79%) |  |
| mnhA3 | Monovalent cation/H+ antiporter, subunit A | NITGRv1_160010 | *Staphylococcus haemolyticus* (Q4L4W7; 803/176, 0.2, 27%) | *Candidatus* Poribacteria sp. WGA-A3 (D2MI05; 176/176, 1.0, 78%) | fragment, part 3 |
| mnhB | Monovalent cation/H+ antiporter, subunit B | NITGRv1_160011 | *Bacillus pseudofirmus* (Q9RGZ4; 144/150, 0.9, 30%) | *Candidatus* Poribacteria sp. WGA-A3 (D2MI06; 150/150, 1.0, 87%) |  |
| mnhC | Monovalent cation/H+ antiporter, subunit C | NITGRv1_160012 | *Methanocaldococcus jannaschii* (Q58706; 128/125, 1.0, 34%) | *Candidatus* Poribacteria sp. WGA-A3 (D2MI07; 125/125, 1.0, 79%) |  |
| mnhD | Monovalent cation/H+ antiporter, subunit D | NITGRv1_160013 | *Desulforudis audaxviator* (B1I6I5; 501/489, 1.0, 36%) | *Candidatus* Poribacteria sp. WGA-A3 (D2MI08; 489/489, 1.0, 74%) |  |
| mnhA1 | Monovalent cation/H+ antiporter, subunit A | NITGRv1_160014 | *Neisseria meningitides* (Q9K1B0; 674/394, 0.5, 44%) | *Candidatus* Poribacteria sp. WGA-A3 (D2MI25; 668/394, 0.5, 83%) | fragment, part 1 |
| mnhA2 | Monovalent cation/H+ antiporter, subunit A | NITGRv1_160015 | *Paracoccus denitrificans* (P29924; 703/366, 0.1, 36%) | *Candidatus* Poribacteria sp. WGA-A3 (D2MI25; 668/366, 0.5, 51%) | fragment, part 2 |
| nuoM | NADH-quinone oxidoreductase, subunit M | NITGRv1_160016 | *Rhodobacter capsulatus* (P50974; 512/490, 1.0, 45%) | *Candidatus* Poribacteria sp. WGA-A3 (D2MI10; 361/490, 0.7, 84%) |  |
| nhaP | Na+/H+ antiporter | NITGRv1_170094 | *Arabidopsis thaliana* (Q9LKW9; 1146/413, 0.4, 28%) | *Desulfatibacillum alkenivorans* (B8FG11; 410/413, 1.0, 62%) |  |
| nhaA | Na+/H+ antiporter NhaA | NITGRv1_270009 | *Pseudomonas putida* (Q88NS2; 397/406, 0.9, 53%) | *Pseudomonas fluorescens* (C3K866; 391/406, 1.0, 49%) |  |
|  | Na+/H+ exchanger | NITGRv1_280116 | *Pseudomonas fluorescens* (Q3KJ66; 580/609, 0.7, 24%) | *Nitrosococcus halophilus* (D5C1F8; 616/609, 1.0, 48%) |  |
| *Mixed defense systems* | | | | | |
|  | Multicopper oxidase | NITGRv1_620037 | *Carboxydothermus hydrogenoformans* (Q3A8Q5; 424/1634, 0.2, 23%) | *Candidatus* Nitrospira defluvii (D8PIB; 1621/1634, 1.0, 55%) | possible manganese oxidase |
| cynS | Cyanate hydratase | NITGRv1_220001 | *Physcomitrella patens* (A9TND9; 154/150, 1.0, 56%) | *Chlorella variabilis* (E1ZMV9; 207/150, 0.7, 57%) |  |
| glpE | Thiosulfate sulfurtransferase | NITGRv1_610029 | *Shewanella oneidensis* (Q8E8J2; 101/107, 0.9, 43%) | *Alteromonadales* bacterium TW-7 (A0XXV6; 118/107, 0.8, 43%) |  |
| arsC | Arsenate reductase | NITGRv1_170075 | *Geobacillus thermodenitrificans* (A4INR2; 140/145, 1.0, 52%) | *Pelobacter carbinolicus* (Q3A3P2; 148/145, 1.0, 67%) |  |
| cld | Chlorite dismutase | NITGRv1_800011 | *Mycobacterium smegmatis* (A0QW25; 231/315, 0.7, 28%) | *Candidatus* Nitrospira defluvii (D8PHP0; 235/315, 0.7, 61%) |  |
|  | Mercuric reductase | NITGRv1_20027 | *Staphylococcus aureus* (P0A0E5; 547/480, 0.8, 36%) | *Aromatoleum aromaticum* (Q5NYA2; 511/480, 0.9, 65%) |  |
| copA | Copper-exporting P-type ATPase A | NITGRv1_590032 | *Bacillus subtilis* (O32220; 802/828, 1.0, 48%) | unc. marine *Nitrospinaceae* bacterium (A4GJ03; 822/828, 1.0, 59%) |  |
|  | Divalent cation tolerance protein CutA | NITGRv1_310077 | *Arabidopsis thaliana* (P93009; 182/108, 0.5, 53%) | *Tribolium castaneum* (D6W8Z0; 116/108, 0.9, 56%) |  |
|  | Cation efflux protein | NITGRv1_140022 | *Xenopus laevis* (Q6GLN7; 281/227, 0.7, 25%) | *Geobacter uraniireducens* (A5G704; 216/227, 0.9, 40%) |  |
|  | Cation efflux protein | NITGRv1_610032 | *Arabidopsis thaliana* (Q8H1G3; 457/305, 0.5, 33%) | *Ferrimonas balearica* (E1SU08; 302/305, 1.0, 44%) |  |
|  | Acriflavin resistance protein | NITGRv1_10012 | *Rhizobium meliloti* (P25197; 1065/1085, 1.0, 26%) | *Cyanothece* sp. CCY0110 (A3IMH8; 1060/1085, 1.0, 36%) |  |
| acrB | Multidrug efflux transporter | NITGRv1_610036 | *Yersinia enterocolitica* (A1JKW9; 1024/1086, 1.0, 26%) | *Planctomyces maris* (A6C4J8; 1185/1086, 1.0, 43%) | Probable acriflavin resistance protein |
| msrA | Peptide methionine sulfoxide reductase | NITGRv1_190010 | *Methanoculleus marisnigri* (A3CUG3; 164/165, 0.9, 63%) | *Cyanothece* sp. strain PCC 7424 (B7K7S5; 162/165, 0.9, 65%) |  |
| msrB | Peptide methionine sulfoxide reductase | NITGRv1_280088 | *Acaryochloris marina* (B0BYW4; 131/134, 0.9, 67%) | *Nitrosospira multiformis* (Q2Y768; 136/134, 0.9, 69%) |  |
|  | Thiol-disulfide interchange protein DsbA | NITGRv1_520009 | *Burkholderia cepacia* (Q9RHV8; 212/207, 0.9, 22%) | *Magnetococcus* sp. strain MC-1 (A0L506; 201/207, 0.7, 29%) |  |
| resA | Thiol-disulfide oxidoreductase | NITGRv1_550029 | *Bacillus thuringiensis* (Q6HL81; 173/189, 0.9, 37%) | *Spirochaeta thermophila* (E0RS23; 226/189, 0.6, 41%) |  |
| ccpA | Cytochrome c peroxidase | NITGRv1_330003 | *Methylobacterium extorquens* (Q49128; 353/334, 0.8, 42%) | *Candidatus* Nitrospira defluvii (D8P897; 365/334, 0.8, 53%) |  |
| trxA | Thioredoxin | NITGRv1_310061 | *Escherichia coli* (P0AA25; 109/110, 1.0, 54%) | *Geobacter metallireducens* (Q39QN1; 109/110, 1.0, 56%) |  |
| trxB | Thioredoxin-disulfide reductase | NITGRv1_680005 | *Arabidopsis thaliana* (Q39242; 383/317, 0.8, 58%) | *Cyanothece* sp. strain PCC 7425 (B8HSJ1; 458/317, 0.7, 61%) |  |
| grxD | Glutaredoxin | NITGRv1_550008 | *Escherichia coli* (P0AC69; 115/108, 0.8, 42%) | *Candidatus* Nitrospira defluvii (D8PJ28; 108/108, 1.0, 74%) |  |
|  | Glutaredoxin | NITGRv1_570008 | *Mus musculus* (Q9CWB7; 115/83, 0.7, 34%) | *Chloroherpeton thalassium* (B3QUM7; 81/83, 0.9, 42%) |  |
| osmC | Peroxiredoxin | NITGRv1_190031 | *Escherichia coli* (P0C0L2; 143/142, 1.0, 53%) | *Herpetosiphon aurantiacus* (A9AZ64; 142/142, 1.0, 63%) |  |
|  | Peroxiredoxin | NITGRv1_360041 | *Dictyostelium discoideum* (Q54SE2; 241/211, 0.9, 61%) | *Candidatus* Poribacteria sp. WGA-A3 (D2MJ52; 211/211, 1.0, 79%) |  |
| bcp | Peroxiredoxin | NITGRv1_550009 | *Coxiella burnetii* (Q83CY8; 151/173, 0.9, 34%) | *Campylobacter upsaliensis* (Q4HQA0; 152/173, 0.9, 39%) |  |
| bcp | Peroxiredoxin | NITGRv1_980003 | *Bacillus subtilis* (Q796Y8; 157/153, 1.0, 56%) | *Planctomyces maris* (A6C1A9; 161/153, 0.9, 62%) |  |

^a^ Organism with highest scoring BLAST hit to *N. gracilis* protein in SwissProt database. In parentheses: SwissProt accession number of best hit; length of SwissProt entry / length of *N. gracilis* protein, alignment length ratio (coverage of longer protein by aligned region of shorter protein), amino acid identity.

^b^ Organism with highest scoring BLAST hit to *N. gracilis* protein in TrEMBL database. In parentheses: TrEMBL accession number of best hit; length of TrEMBL entry / length of *N. gracilis* protein, alignment length ratio (coverage of longer protein by aligned region of shorter protein), amino acid identity.
